# Cefquinome shows a higher impact on the pig gut microbiome and resistome compared to ceftiofur

**DOI:** 10.1186/s13567-023-01176-8

**Published:** 2023-06-06

**Authors:** Sofie Rutjens, Nick Vereecke, Jannes Sauer, Siska Croubels, Mathias Devreese

**Affiliations:** 1 Department of Pathobiology, Pharmacology and Zoological Medicine, Laboratory of Pharmacology and Toxicology, Faculty of Veterinary Medicine, Ghent University, 9820 Merelbeke, Belgium; 2 PathoSense BV, 2500 Lier, Belgium; 3 Department of Translational Physiology, Infectiology and Public Health, Laboratory of Virology, Faculty of Veterinary Medicine, Ghent University, 9820 Merelbeke, Belgium

**Keywords:** Veterinary cephalosporins, gut microbiome, resistome, ARGs, porcine feces, 16S rRNA sequencing, shotgun sequencing, metagenomics

## Abstract

Cephalosporins are licensed for treatment of severe bacterial infections in different species. However, the effect of these antimicrobials on the fecal microbiome and potential spread of resistance-associated genes causes great concern. This highlights the need to understand the impact of cephalosporins on the porcine fecal microbiome and resistome. A combination of long-read 16S rRNA gene and shotgun metagenomic sequencing was applied to investigate the effect of conventional treatment with either ceftiofur (3 mg.kg^−1^ intramuscular, 3 consecutive days) or cefquinome (2 mg.kg^−1^ intramuscular, 5 consecutive days) on the porcine microbiome and resistome. Fecal samples were collected from 17 pigs (6 ceftiofur treated, 6 cefquinome treated, 5 control pigs) at four different timepoints. Treatment with ceftiofur resulted in an increase in Proteobacteria members on microbiome level, while on resistome level selection in *TetQ* containing *Bacteroides*, *CfxA6* containing *Prevotella* and *bla*_*TEM-1*_ containing *Escherichia coli* was observed. Cefquinome treatment resulted in a decline in overall species richness (α-diversity) and increase in Proteobacteria members. On genus level, administration of cefquinome significantly affected more genera than ceftiofur (18 vs 8). On resistome level, cefquinome resulted in a significant increase of six antimicrobial resistance genes, with no clear correlation with certain genera. For both antimicrobials, the resistome levels returned back to the control levels 21 days post-treatment. Overall, our study provides novel insights on the effect of specific cephalosporins on the porcine gut microbiome and resistome after conventional intramuscular treatment. These results might contribute to better tailoring of the most ideal treatment strategy for some bacterial infections.

**Supplementary Information:**

The online version contains supplementary material available at 10.1186/s13567-023-01176-8.

## Introduction

According to the Centers for Disease Control and Prevention (CDC), antimicrobial resistance (AMR) will cause approximately 10 million annual deaths by 2050, making it one of the top 10 threats to global health [1, 2]. Although the increase in AMR was seen even before the introduction of antimicrobials, there is a general agreement that the increase in AMR is strongly related to the use and especially the misuse of antimicrobials [3]. Concurrent use of antimicrobials in both humans and animals drives the emergence and transmission of acquired resistance only further [4]. The World Health Organization (WHO) has made a ranking of antimicrobials that are of critical importance to human medicine. The prudent use of these antimicrobials, especially in veterinary medicine, should help to preserve the efficacy of the available antimicrobials. Quinolones, third- and fourth-generation cephalosporins, macrolides and ketolides, and glycopeptides are considered antimicrobials of highest priority [5]. Cephalosporins belonging to the 3rd and 4th generation (e.g. ceftriaxone, cefodizine, cefcapene, etc.) represent antimicrobials often used as a last resort to treat severe, invasive infections in human medicine [6, 7]. Consequently, the administration of 3rd and 4th generation cephalosporins in veterinary medicine is under scrutiny [8]. Though, their overall use in veterinary medicine is low [9]. Since, the fear of potential ineffectiveness of cephalosporins in human medicine resulted in a voluntary ban on their veterinary use in some countries, which resulted in a reduction in associated resistance levels [10, 11]. Still, third- and fourth-generation cephalosporins were shown to be the sole effective pharmacotherapy against some veterinary pathogens (e.g. treatment of foal septicaemia caused by *Escherichia coli*). Banning these antimicrobials could, therefore, result in increased animal suffering and decreased welfare [12–14]. In pig production, these cephalosporins are administered for the treatment of post-partum dysgalactia syndrome, respiratory diseases and *Streptococcus suis* infections [13, 15]. To date, alternatives, such as less critical antimicrobials can often still be administered to treat these infections in pigs as limited acquired AMR has been observed [13]. However, it is of utmost importance to gain better understanding of these cephalosporins and their impact on AMR dissemination after administration to rationalize their use.

Third- and fourth-generation cephalosporins are administered intramuscularly because of their low oral bioavailability. Interestingly, this route of administration prevents direct exposure of the gastro-intestinal tract to these antimicrobials, however, after absorption they may partly get excreted into the intestinal tract [16]. Studies have confirmed the selective effect on resistance of these third- and fourth-generation cephalosporins on the gut microbiome, and especially on the resistance selection in *Escherichia coli* [17, 18]. These findings raised concern on the spread of associated resistance genes found in commensal bacteria of animals to pathogenic bacteria in humans via food and the environment [19]. Judicious use of antibiotics requires not only knowledge on the impact of the treatment on target pathogens or indicator bacteria, such as *Escherichia coli*, it is also important to obtain knowledge on the effect on the overall gut microbiome and changes in its associated resistome. To date, little information on this complex matter is available. Some studies provide data on the effect of the administration of a single dose, however, no information is available on the impact of a full relevant treatment schedule on the microbiome and resistome of pigs [20–22]. This lack of information limits the possibility for well-founded treatment decisions and administration schemes that take dissemination of AMR-associated genes into account, as well as potential discrepancies between different cephalosporins.

Building on our previous preliminary findings, a combinational metagenomic analysis was applied to determine the effect on the microbiome and resistome after a full treatment schedule with two important veterinary cephalosporins, ceftiofur and cefquinome [16]. Here, long-read nanopore 16S rRNA gene sequencing and associated diversity and composition analyses were performed. This was combined with long-read nanopore shotgun sequencing to study the differential abundance in antimicrobial resistance genes (ARGs). Together the results of these two analyses delivered a well-founded overview of the effect of these antimicrobials on the porcine gut microbiome and resistome. The study design included a control group, to adjust the results for age-related changes in the microbiome and resistome.

## Materials and methods

### Animal trial

The animal trial was approved by the ethical committee of the Faculty of Veterinary Medicine and of Bioscience Engineering of Ghent University (EC 2021-107). Animal care complied with the Belgian and European legislation on animal welfare and ethics [23, 24].

A group of 17 healthy, stress-resistant 7-week-old pigs (9 male, 8 female, Landrace × Large White × Maximus, Seghers Hybrid^®^, Wuustwezel, Belgium) of 18.0 kg ± 1.4 (standard deviation) kg body weight, were separated into two groups of 6 pigs and one group of 5 pigs. Each group was housed in a different adjacent stable and each pig was housed individually to prevent contamination and coprophagy between the groups and different pigs. The pigs had ad libitum access to water and were fed twice daily (Biggispeen Premium^®^, Aveve, Leuven, Belgium). Before the start of the treatment, the pigs were acclimatized for 10 days.

**Figure 1:**
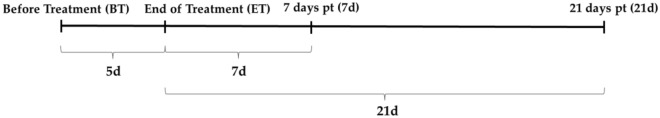
**Timeline of the four sampling points during the animal trial**.

Group one, consisting of six pigs (three male, three female) received an intramuscular injection in the biceps femoris of cefquinome at 2 mg.kg^−1^ body weight at the same timepoint on 5 consecutive days, in accordance with the leaflet (Ceffect^®^, Emdoka, Hoogstraten, Belgium). The second group of six pigs (three male, three female) was administered 1 mL of a physiological saline solution at the same timepoint as group one on the first 2 days, to mimic treatment handling. The following 3 days they received an intramuscular dose in the biceps femoris of ceftiofur at 3 mg.kg^−1^ body weight at the same timepoint, in accordance with the leaflet (Excenel flow^®^, Zoetis, Louvain-la-Neuve, Belgium). Group three, the control group, consisted of five pigs (three male, two female) and received an intramuscular injection in the biceps femoris with 1 mL of a physiological saline solution for 5 consecutive days. Fresh feces (1 g) of each pig was sampled by collection of fresh fecal droppings in a cleaned stable on the morning before the start of the 5 day treatment period (BT), on the evening on the last day of treatment (ET), and on the morning at one and three weeks (7d and 21d) post-treatment (pt). The center of the dropping was subsampled to ensure the accuracy of the bacterial composition. Figure 1 depicts the sampling timeline during the animal trial. The fecal samples were stored at ≤  −70 °C until analysis.

### DNA extraction, library preparation and sequencing

DNA extraction was performed on 250 mg feces using the QIAamp PowerFaecal Pro DNA Kit (QIAGEN, Antwerp, Belgium) as per manufacturer’s instructions. DNA concentration and purity was measured by evaluating the A_260/280_ and A_260/230_ absorbances on a Nanodrop spectrophotometer. All samples were subjected to an additional magnetic bead clean-up using CleanNGS (CleanNA, Waddinxveen, the Netherlands). The cleaned DNA was subsequently subjected to targeted 16S rRNA gene amplification and shotgun sequencing to identify the bacterial diversity and relative abundance, and the presence of AMR genes, respectively. The integrity of the 16S rRNA gene fragments was visually validated with gel electrophoresis. For 16S rRNA gene and shotgun sequencing library preparations, the SQK-16S024 and SQK-RBK110-96 were used, respectively (ONT; Oxford, UK). In both instances manufacturer’s instructions were followed. Finally, the libraries, containing 24 and 12 barcoded samples per library, respectively, were loaded onto a R9.4.1 flow cell prior to sequencing on a GridION sequencer for 36 h.

### Sequenced data processing and statistical analysis

After sequencing of the 16S rRNA gene fragments, classification was done using the state-of-the-art emu software [25, 26]. Base calling and demultiplexing was performed in real-time using the “super accurate” base calling model in Guppy (v6.2.7; ONT) and were quality filtered using NanoFilt (v.2.7.1; [27]). The reads were classified to their closest matching hit and classified up to species level. Filtering criteria were determined based on the overall number of classified reads. A threshold of 7500 classified reads was set to assign phylum, family, or genus. The raw data resulting from the shotgun sequencing were taxonomically classified using Kraken2 (v.2.0.9; k2_pluspf_20210127; [28]). These taxonomically binned reads where then used to independently search for both ARGs and plasmid replicons using the Comprehensive Antimicrobial Resistance Database (CARD) [29] and PlasmidFinder [30] respectively. Actual screening was performed using Abricate (v1.0.1) [31] with default settings and at a minimum coverage and nucleotide identity of 80% and 60%, respectively. Final ARG abundances were generated per bacterial taxon and per sample as well as identifying reads harbouring both gene hits for plasmid replicons and ARGs. The raw sequencing datafiles are available on the European Nucleotide Archive (ENA) under project PRJEB61541 and accessions ERS1494721-ERS14949789 (Additional file 1).

For 16S rRNA gene data, the α-diversity was analyzed based on the Shannon index (SI) using genus-level classifications. For the statistical comparison of the diversity index (Shannon index) between the different sampling points of each group, a Kruskal–Wallis test was done followed by a Wilcoxon rank sum test. Log_2_ fold changes in abundance (of 16S rRNA classified genera and shotgun ARGs) within each treatment group (16S rRNA) or between each treatment group and the control group (shotgun ARGs) at a given sampling point were determined by the negative binomial generalized linear model in the DESeq2 package (V1.32.0) in R (V4.1.2) [32]. The corresponding heatmap of the Log_2_ fold changes of the genera was visualized by using the Heatmap function in the R package complexHeatmap (V2.8.0) [33]. The permutational multivariate analysis of variance in microbiome and resistome was quantified by pairwise comparison of the Bray–Curtis dissimilarity matrices, using the adonis2 function in the R package vegan (V2.6.2) [34]. A corresponding Principal Coordinates Analysis (PCoA) was visualized with the ggplot2 package (V3.3.6) and a correlation-coefficient analysis was performed for each sampling point using the envfit function in the vegan package [35]. To avoid loss of potentially interesting results, statistical significance was tested at corrected *P*-values ≤ 0.10 (Bonferroni correction—Shannon index, FDR-correction—other statistics).

## Results

16S rRNA sequencing of the 68 porcine fecal samples resulted in an average of 81 559 (± 18 650) reads with an N_50_ length of 1484 (± 61) nucleotides (Additional file 1). Whilst shotgun sequencing resulted in an average of 241 326 (± 121.660) reads with an N_50_ length of 4023 (± 893) nucleotides (Additional file 2). Based on the rarefaction curves of the 16S rRNA gene sequencing, a cut-off of 7500 reads was set, which resulted in a satisfactory taxon richness for all samples (Additional file 3).

### Impact on diversity

**Figure 2:**
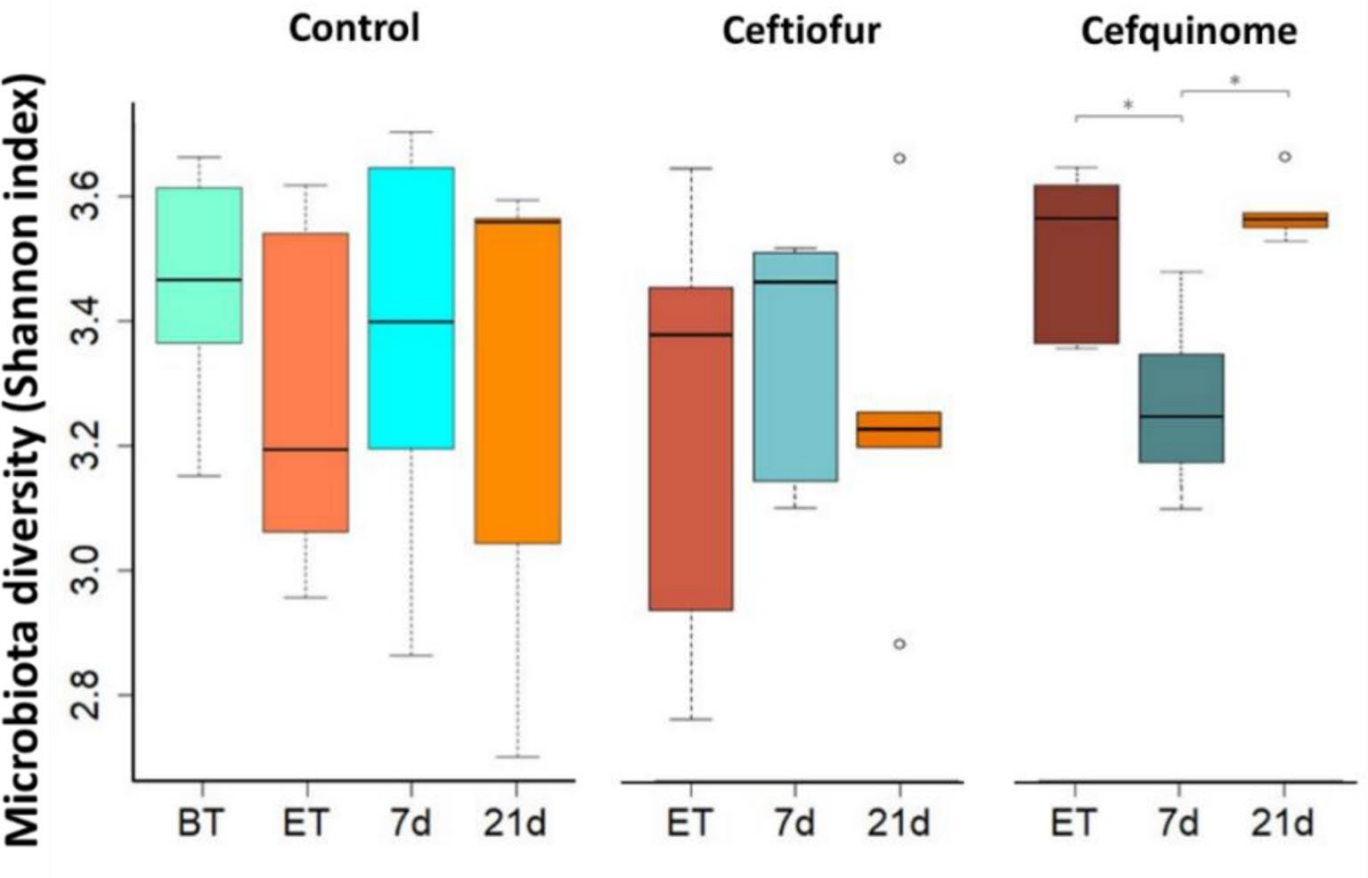
**Alpha diversity analysis (Shannon Index) of the fecal porcine microbiome at genus level.** Box-plots of the Shannon index are shown. *Bonferroni-corrected *P*-value ≤ 0.10. Following either ceftiofur treatment: 3 mg.kg^−1^ intramuscular, 3 consecutive days or cefquinome treatment: 2 mg.kg^−1^ intramuscular, 5 consecutive days. (BT = Before-treatment (n = 17), ET = End of Treatment, 7d = 7 days post-treatment, 21d = 21 days post-treatment).

The Shannon index showed that the evenness of the fecal microbiota in the animals of the control group fluctuated over time, though no significant alterations were identified at any of the different timepoints. For the animals receiving ceftiofur, a comparable fluctuation could be observed. While an apparent decrease in SI was observed at 21 days post-treatment (dpt) for the ceftiofur treated group, no statistical significance was observed. For the animals of the cefquinome treatment group, a significant decline in SI was detected between the ET (SI 3.5229 ± 0.1375) and 7 dpt samples (SI 3.2478 ± 0.1519, Bonferroni-corrected *P*-value 0.026), which returned back to the initial end of treatment levels by 21 dpt (SI 3.5825 ± 0.0514, Bonferroni-corrected *P*-value 0.006) (Figure 2).

### Impact on taxonomic profiling

**Figure 3:**
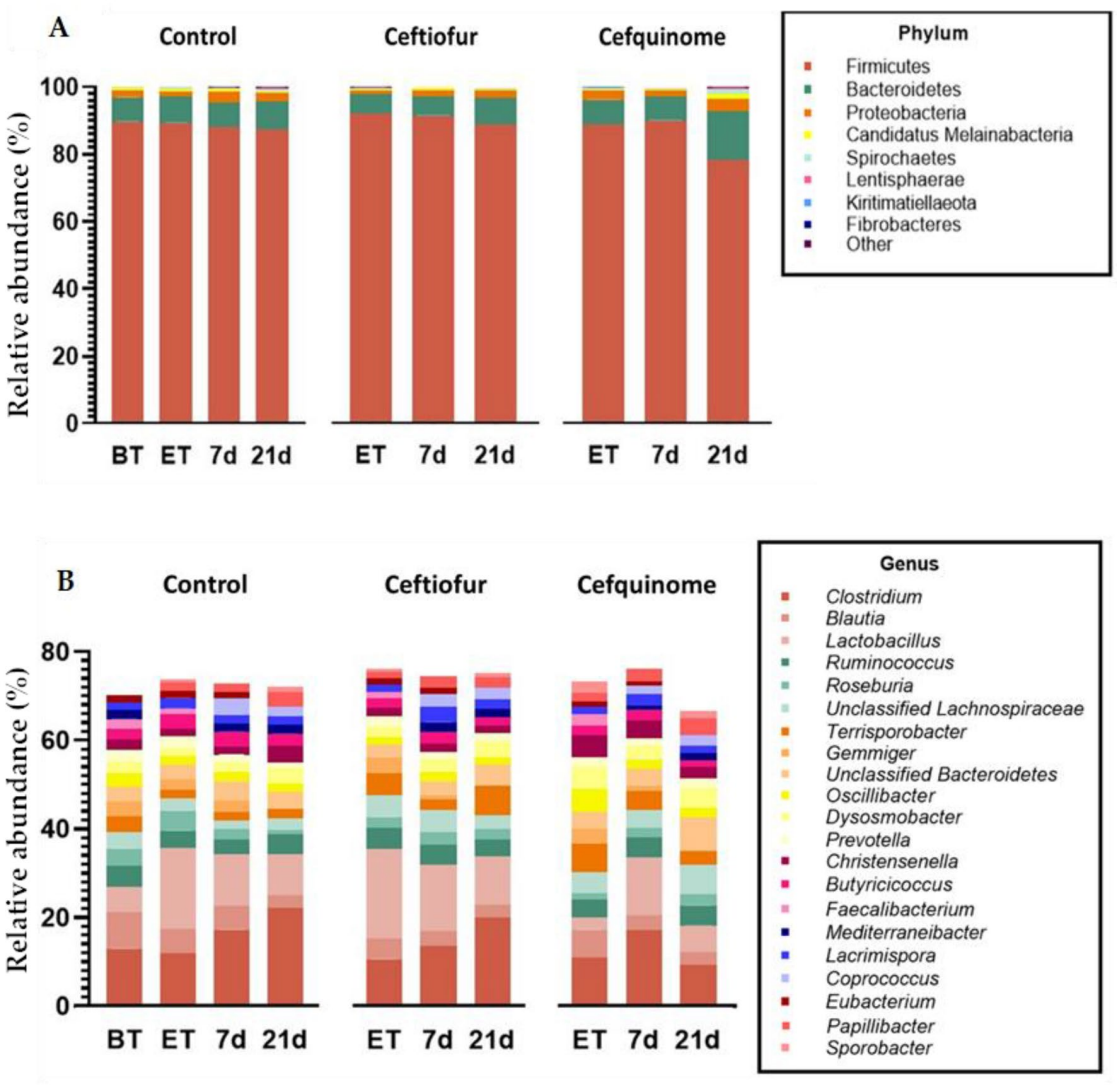
**Phylum and genus abundances. A** Mean relative abundances at the phylum level in the porcine fecal microbiome based on 16S rRNA gene analysis. **B** Top 21 most abundant genera in the porcine fecal microbiome based on 16S rRNA gene analysis. Following either ceftiofur treatment: 3 mg.kg^−1^ intramuscular, 3 consecutive days or cefquinome treatment: 2 mg.kg^−1^ intramuscular, 5 consecutive days. (BT = Before-treatment, ET = End of Treatment, 7d = 7 days post-treatment, 21d = 21 days post-treatment).

The taxonomic profiles of the fecal samples for each sampling point per group is visualized in Figure 3. At the phylum level, Firmicutes was distinctly the most abundant phylum in the porcine microbiome, comprising almost 90% of the total microbial community (Figure 3A). The other 10% consists mainly of Bacteroidetes and Proteobacteria. In the cefquinome group a decrease in abundance of Firmicutes accompanied by an increase in Bacteroidetes was observed at 21 dpt, which did not occur in the control or ceftiofur group. On genus level (Figure 3B), *Clostridium* was the most abundant genus before the start of the antimicrobial treatment, followed by *Blautia* and *Lactobacillus*. The genus composition at the end of treatment is comparable between the control group and the ceftiofur group. However, in the cefquinome group, a smaller amount of *Lactobacillus* was measured, while the levels of *Christensenella* and *Oscillibacter* were relatively higher as compared to the control and ceftiofur group at this timepoint. Over time an increase in *Clostridium* could be observed in the control group, which could also be observed in the ceftiofur group. For cefquinome, this increase could be observed at the first two sampling points, but was followed by a drop at 21 days post-treatment.

**Figure 4:**
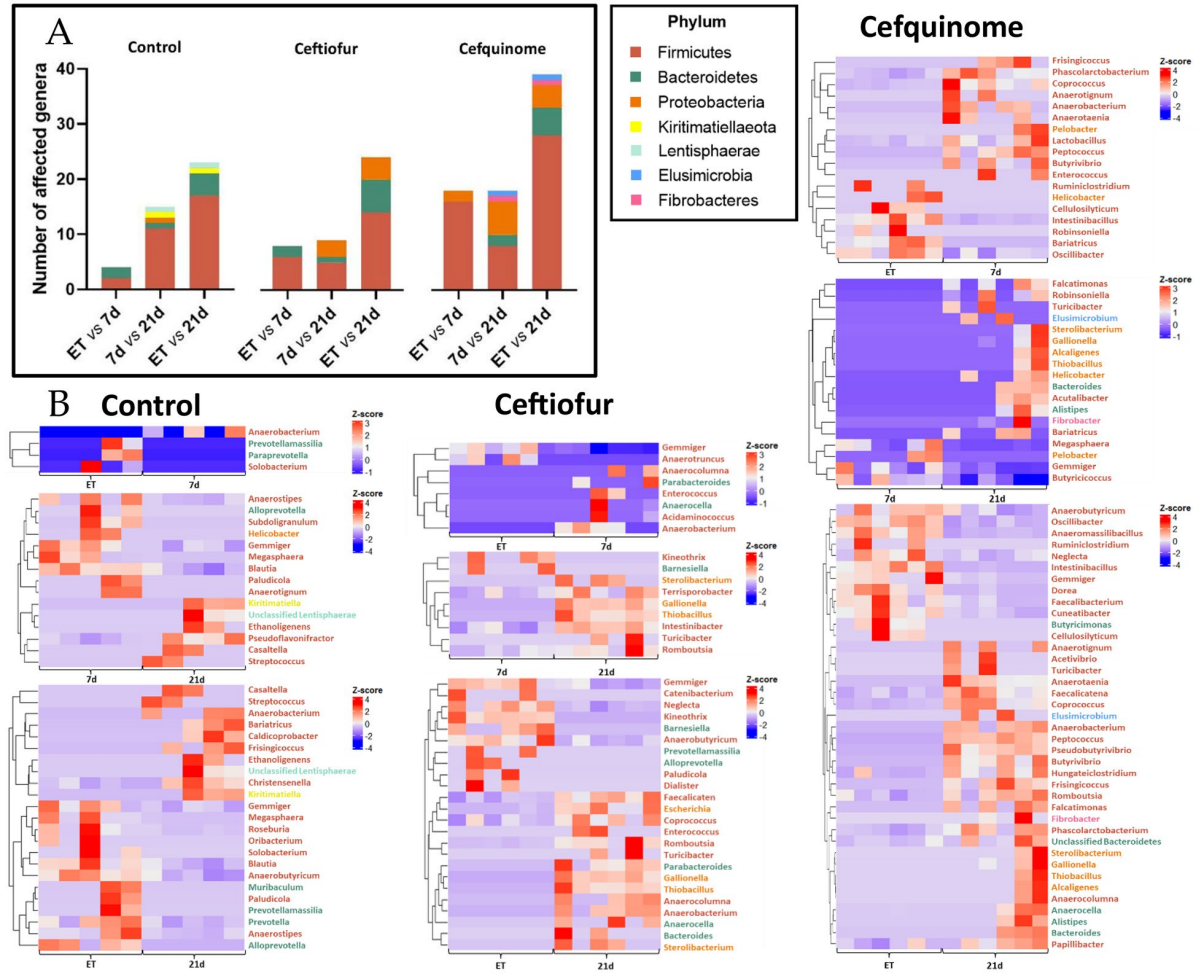
**Impact of time and treatment on the differential abundances of the genera in the porcine fecal microbiome. A** Bar plot showing the number of affected genera over the course of the study.** B** Heatmaps showing the change in affected genera between the different time points in each studied group and colour labelled according to their corresponding phylum. Changes following either ceftiofur treatment: 3 mg.kg^−1^ intramuscular, 3 consecutive days or cefquinome treatment: 2 mg.kg^−1^ intramuscular, 5 consecutive days. Red shades indicate an increase in abundance, while blue shades indicate a decrease. (BT = Before-treatment, ET = End of Treatment, 7d = 7 days post-treatment, 21d = 21 days post-treatment).

Differential abundance analyses of the taxonomic assignments at the genus level were performed using DESeq2. This showed a significant impact of the antimicrobial treatments on the porcine fecal microbiome during the studied sampling period. Genera belonging to the Firmicutes phylum were most affected by both time and treatment, followed by Bacteroidetes and Proteobacteria (Figure 4A, Additional file 4). Of interest is the remarkable impact on Proteobacteria following both ceftiofur and cefquinome treatment, which is not detected in the control group. However, in the control group Kiritimatiellaeota and Lentisphaerae belong to the affected phyla, which is not the case in the treatment groups. Following cefquinome treatment, some samples show an increase in Elusimicrobia at 21 dpt. On genus level it could be observed that in both control and ceftiofur group at 7 dpt the significant impact was limited to a small number of genera. Four genera were affected because of time, and eight were affected because of time and ceftiofur treatment. More important, for cefquinome 18 genera were affected by time and antimicrobial administration at 7 dpt, with a significant (FDR-corrected *P*-value < 0.10) increase in the abundance of 11 genera, of which 10 belonged to the Firmicutes phylum. Also, a significant reduction of seven genera was observed, of which six belonged to the Firmicutes phylum (Figure 4B). In general, the number of affected genera between 7 dpt samples and the 21 dpt samples remained similar to the affected number between the end of treatment and 7 dpt samples.

### Microbiome profile variations

**Figure 5:**
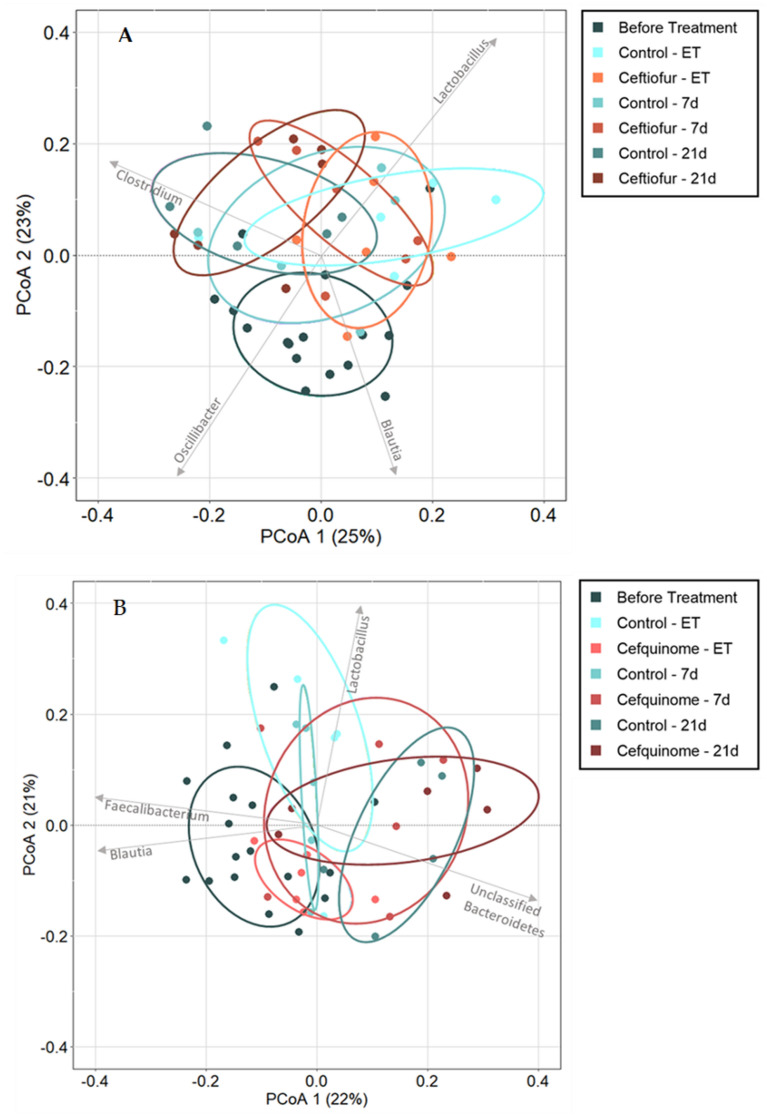
**Principal Coordinates Analysis (PCoA) using the Bray–Curtis dissimilarity index showing changes in microbiome profiles. A** Control group and ceftiofur group. **B** Control group and cefquinome group. The arrows depict a significant influential genus per quadrant of the graph. Following either ceftiofur treatment: 3 mg.kg^−1^ intramuscular, 3 consecutive days or cefquinome treatment: 2 mg.kg^−1^ intramuscular, 5 consecutive days. (ET = End of Treatment, 7d = 7 days post-treatment, 21d = 21 days post-treatment).

Permutational multivariate analysis of variance using the Bray–Curtis distance matrix indicated that the combination of time and antimicrobial administration could significantly alter the profile of the relative abundance of genera on all sampling points in comparison to the profile in the blank samples (i.e. samples taken of all pigs before treatment) (Additional file 5: Blank vs ET_CT, etc.; FDR-corrected *P*-value < 0.10). This is in line with the minimal overlap of the before-treatment samples with all other sampling points displayed on the PCoA’s (Figures 5A, B). However, for ceftiofur no significant alteration could be observed between the samples of the ceftiofur group and the control group at the same timepoints (ET_Cont vs ET_CT, etc.) suggesting that the observed difference between the before-treatment levels and the levels at the different sampling points after ceftiofur treatment is mainly influenced by time. This is visualized by the consistent overlap of the control samples and ceftiofur samples for each sampling point. Interestingly, the data did indicate a significant impact of cefquinome immediately at the end of treatment (ET) (Additional file 5: Adonis R2 0.3498, FDR-corrected *P*-value 0.0036), which is in line with the separation of the samples belonging to either the cefquinome group or the control group at the end of treatment displayed on the PCoA plots (Figure 5B).

Correlation-coefficient analysis of the top 21 most abundant microbial genera resulted in a list of influential genera that drive the observed divergence among the different datapoints (Additional file 6). For each quadrant, a significant influential genus based upon the R^2^ value has been depicted on the PCoA (Figure 5). For ceftiofur, *Lactobacillus* and *Clostridium* were the main drivers (R^2^ 0.9180 and 0.8083, respectively), followed by *Blautia* and *Oscillibacter*. In the cefquinome treatment group, mainly *Lactobacillus* and *Blautia* were drivers of divergence (R^2^ 0.7496 and 0.6926, respectively). The observations in both groups are in line with the change in relative abundance (Figure 3B), where a remarkable increase in *Lactobacillus* and a small decrease in *Blautia* between the blank samples and the samples taken during the study was shown. However, in the cefquinome group, *Papillibacter* exhibited the strongest correlation (R^2^ 0.7606) but is thought to be less important due to its low level of abundance within the microbial community (± 1%).

### Impact on antibiotic resistome profiling

**Figure 6:**
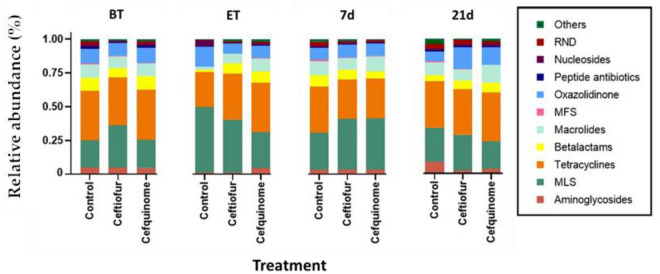
**Relative abundance of the top 10 classes of antibiotics to which most ARGs in porcine fecal samples are directed against, during the study**. Following either ceftiofur treatment: 3 mg.kg^−1^ intramuscular, 3 consecutive days or cefquinome treatment: 2 mg.kg^−1^ intramuscular, 5 consecutive days. (BT = Before-treatment, ET = End of Treatment, 7d = 7 days post-treatment, 21d = 21 days post-treatment).

Antibiotic resistome profiling of the porcine fecal samples depicted that, regardless of the treatment with antibiotics, all samples contained a diverse, but comparable set of ARGs (Figure 6). The two most predominant classes of ARGs were directed to either tetracyclines (mean = 33.64 ± 8.98 throughout the study) or macrolide-lincosamides-streptogramins (MLS) (mean 30.24 ± 14.98 throughout the study), followed by ARGs against oxazolidinone and macrolides alone.

Differential abundance analysis revealed a significant increase (FDR-corrected *P*-value ≤ 0.10) in ARGs in the samples but only immediately at the end of the treatment (Table 1). In both the ceftiofur and cefquinome group, ARGs directed against tetracyclines (*tetQ* and *tet(40*)) showed an increase in abundance. However, for cefquinome, several other ARGs showed an increase in abundance, such as *lsaB* (multi-drug ABC efflux pumps), *ErmF* (23S rRNA methyltransferases), *CfxA6* (Class A beta-lactamases) and *mel* (Macrolides Lincosamides Streptogramines resistance Major Facilitator Superfamily efflux pumps). The most strongly increased ARGs in the cefquinome treated group were *mel* and *tetQ* with an approximately 2.5 log_2_ fold change. Important to note, no ARGs showed a decrease in abundance after the administration of either cefquinome or ceftiofur. Furthermore, our data suggested that within one week after cessation of the antibacterial treatment, the ARG resistome recovered to the same level as the control group, as no significant differential abundance in ARGs between the control group and either the ceftiofur or cefquinome group could be observed.

**Table 1:** **Differential abundance analysis of the antibiotic resistance genes in the porcine fecal samples**

	ARG	baseMean	Log_2_Foldchange	q-value	Mechanism
Ceftiofur	*tetQ*	6.27	2.14	0.05	Tetracycline resistance ribosomal protection proteins
Cefquinome	*mel*	8.51	2.58	0.00	MLS resistance MFS efflux pumps
	*tetQ*	8.33	2.54	0.00	Tetracycline resistance ribosomal protection proteins
	*ErmF*	7.81	2.43	0.00	23S rRNA methyltransferases
	*CfxA6*	4.21	2.16	0.03	Class A beta-lactamases
	*lsaB*	7.71	1.76	0.02	Multi-drug ABC efflux pumps
	*tet(40)*	7.37	1.68	0.03	Tetracycline resistance ribosomal protection proteins

Comparison of samples collected immediately after treatment (ET) with either ceftiofur (3 mg.kg^−1^ intramuscular, 3 consecutive days) or cefquinome (2 mg.kg^−1^ intramuscular, 5 consecutive days) to those collected from the control group at the same timepoint.

### Resistome profile variations

**Figure 7:**
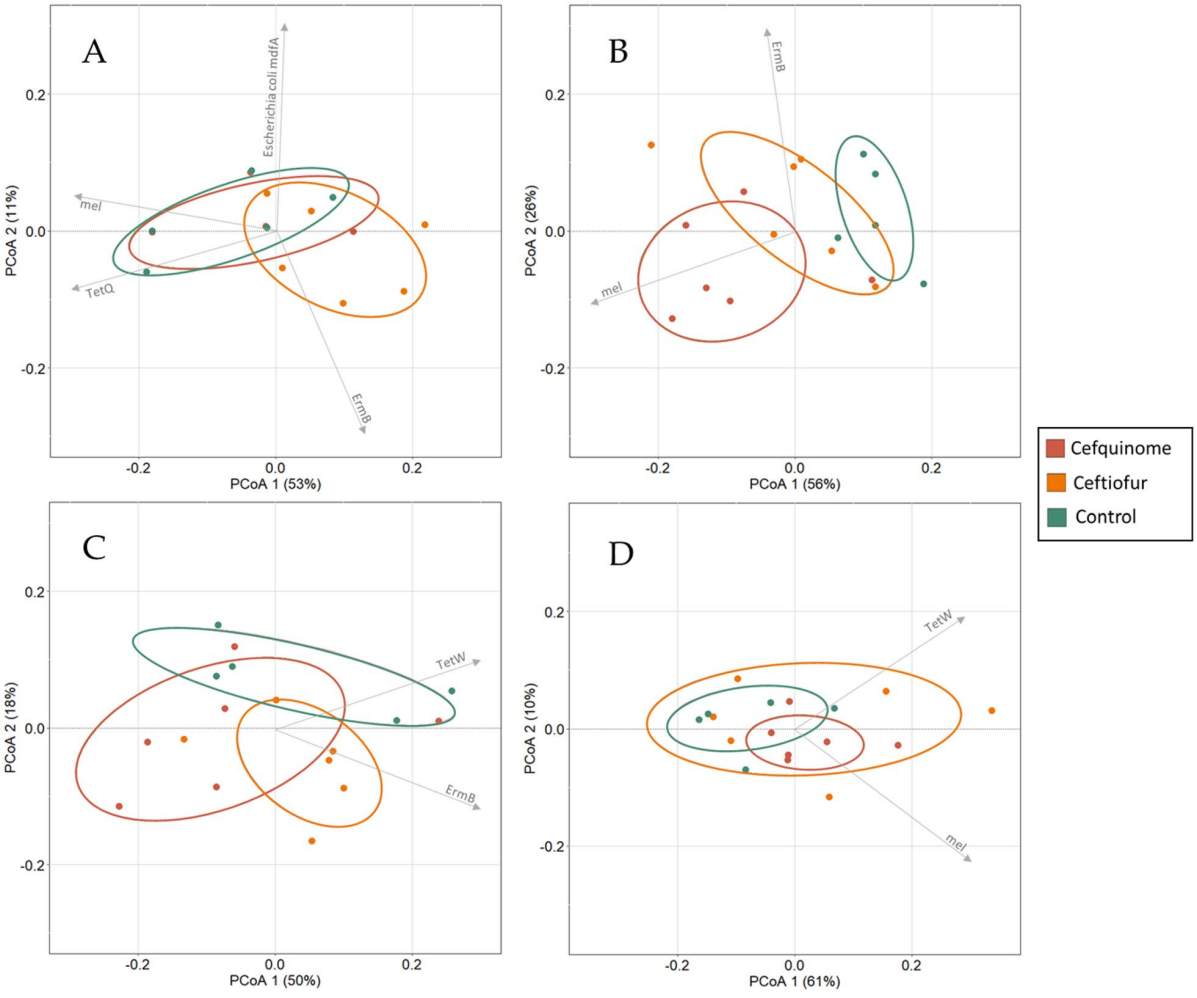
**Principal Coordinates Analysis (PCoA) using the Bray–Curtis dissimilarity index showing the change in resistome profile. A** Change in resistome profile before-treatment. **B** Change in resistome profile at the end of treatment. **C** Change in resistome profile 7 days post-treatment. **D** Change in resistome profile 21 days post-treatment. The arrows depict a significant influential ARG with R2 > 0.4 at the observed timepoint for that quadrant (if present). Following either ceftiofur treatment: 3 mg.kg^−1^ intramuscular, 3 consecutive days or cefquinome treatment: 2 mg.kg^−1^ intramuscular, 5 consecutive days.

Permutational multivariate analysis of variance using the Bray Curtis distance matrix indicated a significant variation of 45.50% in profile of ARGs counts at the end of treatment (Adonis R^2^, FDR-corrected *P*-value 0.0720) that could be explained by cefquinome treatment (ET_Cont vs ET_CQ) (Additional file 7). This is in agreement with the corresponding PCoA which displays a clear separation of the samples collected from the cefquinome group as compared to the control group (Figure 7B). On the other hand, animals that received ceftiofur showed no significant impact on the ARG counts profile at the end of treatment (Adonis R2 0.2257, FDR-corrected *P*-value 0.1898). For cefquinome, the observed difference could no longer be detected in the samples taken 7 days and 21 days pt. However, at 7 dpt a wide dispersion of the samples from the cefquinome group could still be observed (Figure 7C). Although the overall effect of cefquinome treatment on that timepoint was not significantly different than in the control group, this dispersion indicated that cefquinome administration potentially contributed to a wide variety of effects, which might be animal dependent. At 21 dpt, the antimicrobial resistome profile of all three groups was comparable again (Figure 7D).

Correlation-coefficient analysis of the different sampling points resulted in a list of influential antimicrobial resistance genes that drove the observed divergence among datapoints (Additional file 8, R^2^ > 0.4 cut-off). Before-treatment the most influential ARGs were *tetQ* and *tetW* (tetracycline resistance ribosomal protection proteins), *CfxA6* (Class A beta-lactamase) and *lsaB* (Multi-drug ABC efflux pump). Immediately after treatment cessation and up until 7 dpt, *ErmB* (23S rRNA methyltransferase) became the most influential ARG, mainly driving the ceftiofur group, which is also visualized in Figure 7B and Figure 7C. Interestingly, *ErmB* was shown to be plasmid-borne in both *Lactobacillus* and *Limosilactobacillus* species. In the case of *Lactobacillus*-associated *ErmB*, the same repUS69 replicon (with closest match to the pRKC30SC1 plasmid (CP002560)) was identified on the same read for all the samples. Also for three *Limosilactobacillus* classified read bins, a match with the repUS69 replicon was found, but now with a closest match to the pTE44 plasmid (AY082384). Cefquinome divergence was driven by *mel* both at end of treatment and at 21 days post-treatment. The importance of *mel* as a driving force within the cefquinome group is in line with the results obtained from the differential abundance analysis. Finally, only two other ARG-encoding plasmids were identified in current dataset, including the *tetK* and *mcr4.2* genes in *Staphylococcus* (sample 1–0004918) and *Escherichia* species (sample 1–0004930). These were present on a plasmid carrying a rep7a replicon cassette (AB037671) and ColE10 replicon (X01654), respectively.

### Antimicrobial resistance genes—genus allocation

**Figure 8:**
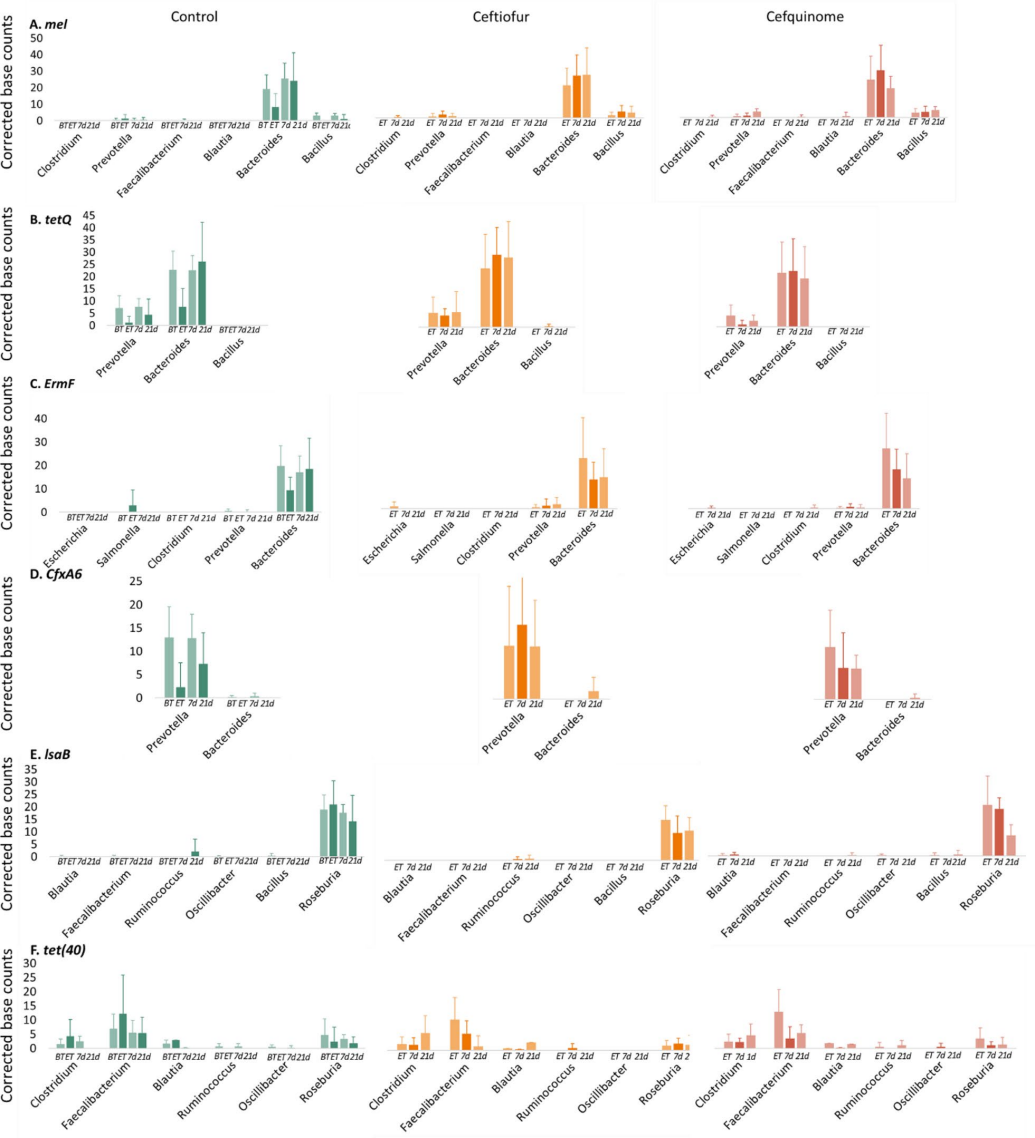
**Mean resistance gene counts corrected to the base counts of the six resistance genes.** Bar plots (+ standard deviation) showing the mean resistance gene counts with a significant log_2_ fold change of each sample corrected to the base counts following either ceftiofur treatment: 3 mg.kg^−1^ intramuscular, 3 consecutive days or cefquinome treatment: 2 mg.kg^−1^ intramuscular, 5 consecutive days. (BT = Before-treatment, ET = End of Treatment, 7d = 7 days post-treatment, 21d = 21 days post-treatment).

Figure 8 depicts an overview of the changes in resistance genes that resulted in a significant log_2_ fold change. The advantage of long-read shotgun sequencing is the possibility to allocate the observed ARGs to a corresponding genus. In order to interpret the results properly, an assumption was made that samples resulting in lower or higher base counts, still represented a homogenous microbial environment. Also, the ARGs counts were corrected to 1.000.000.000 base-counts per sample. The most strongly increased ARG in the fecal samples of the cefquinome treatment group was *mel*, which is mainly associated with *Bacteroides* in all groups. Figure 8A depicts a decrease of *mel* in the cefquinome group at 21 dpt, while the relative abundance of *Bacteroides* in the shotgun sequencing dataset remained stable during the entire treatment period (Additional file 9). For ceftiofur, this decrease was not observed, while also the relative abundance remained stable. In the control group at the end of the treatment sampling point (ET) there was a decrease in resistance genes allocated to *Bacteroides* (Figures 8A, B and C) and *Prevotella* (Figures 8B and D). This general decrease is in line with the decline in relative abundance of these two genera (shotgun data, Additional file 9) at the corresponding sampling point and can thus be explained by normal fluctuations. For cefquinome and ceftiofur, no such decline in relative abundance (shotgun data, Additional file 9) could be observed between the before-treatment levels and those at end of treatment. In contrast, the 21 dpt sampling point in the control group showed overall high ARG counts, while the relative abundance was lower in comparison to the before-treatment sampling point, especially for *Prevotella*. This suggested beneficial properties over time of *Bacteroides* and *Prevotella* harbouring ARGs in contrast to these genera without the ARGs even without the exposure to an antimicrobial. The second important resistance gene, *tetQ*, was associated with both *Prevotella* and *Bacteroides* (Figure 8B). For *tetQ* associated with *Prevotella* the ARG counts followed the changes in relative abundance, so treatment and/or time did not impact the *tetQ*-*Prevotella* ratio. However, for *tetQ* associated with *Bacteroides*, the data suggested a potential selective effect caused by ceftiofur treatment, since here a comparable relative abundance of *Bacteroides* on the 7 dpt sampling point and the before-treatment sampling point was observed. Though, an increase in corrected base counts for *tetQ* was also seen. In the cefquinome treatment group, the fluctuations of *tetQ* followed those of the relative abundance of *Bacteroides*. The third resistance gene, *ErmF* (Figure 8C), was associated with *Bacteroides* and followed the relative abundance fluctuations in all the groups. The next ARG, *CfxA6*, a class A beta-lactamase associated gene, was in this study mainly associated with *Prevotella* and showed no change after cefquinome treatment, since there was both a decrease in ARG counts as in relative abundance of *Prevotella* (Figure 8D). In contrast, in the ceftiofur group, especially on 7 dpt, there was a noticeable increase in *CfxA6* counts, while the relative abundance of *Prevotella* was comparable to that of the other groups. This suggested a selection of *Prevotella* harbouring *CfxA6* after ceftiofur treatment. The next ARG that exhibited a significant log_2_ fold change is *lsaB*, which was associated with *Roseburia* (Figure 8E). The most remarkable change could be seen in the cefquinome group, where a decrease in *lsaB* counts between the 7 days and 21 dpt sampling point was seen, with no apparent decrease in relative abundance. The last resistance gene was *tet(40)* and was mainly associated with *Faecalibacterium*, *Roseburia*, and *Clostridium* (Figure 8F). The changes of *tet(40)* in *Faecalibacterium* and *Roseburia* could be attributed to normal fluctuations, since for all groups the same patterns could be detected. For *tet(40)* on *Clostridium* there was a small increase of ARG counts between 7 and 21 days post ceftiofur treatment, while the relative abundance between these two days remained stable. This suggested a possible selection of *tet(40)* associated *Clostridium* after ceftiofur treatment. The long-read shotgun sequencing data resulted in another interesting finding in the ceftiofur treatment group. In this group an increase of a plasmid-mediated beta-lactamase, *bla*_*TEM-1*_, associated with *Escherichia* could be observed mainly on day 7 but also on day 21 pt, on the other two timepoints no *bla*_*TEM-1*_ was detected.

## Discussion

In this study, we assessed the impact of the administration of either ceftiofur or cefquinome (as instructed on the manufacturers’ leaflet) on the pig fecal microbiota and associated resistome over time by applying both 16S rRNA gene and shotgun sequencing.

We report no significant alterations in the evenness of the α-diversity (Shannon index) in the control and ceftiofur group [36]. In the human gut microbiota an increase in bacterial diversity from birth up to approximately 3 years has been reported, whereafter it reaches a fluctuating but relatively stable status [37]. The same has been observed in micro-pigs and conventional pigs, where a stable community is established approximately 2–3 weeks post-weaning [38, 39]. The reported non-significant changes in α-diversity observed in our study could, therefore, be attributed to normal fluctuations. For cefquinome, a significant decrease in α-diversity was observed in the samples collected 7 dpt followed by a recovery to the initial levels measured at the end of treatment by 21 dpt. However, these levels were still within the same range as for the control and ceftiofur group and are, therefore, considered to be of no notable importance.

Concerning the fecal microbiome, our study indicated that approximately 90% of the observed genera belonged to the Firmicutes phylum on all sampling days (prior and post-treatment). This is in line with the previously observed abundance of Firmicutes in the gut microbiome during the growing phase of pigs [38, 40, 41]. Firmicutes bacteria can ferment carbohydrates to short-chain fatty acids, which play an important role in the intestinal barrier function and are thus essential members of the gastro-intestinal tract of mammals [42]. However, the dominance of Firmicutes competes with that of Bacteroidetes, which was the most abundant phylum in several other studies [43, 44]. These observed discrepancies in the pig fecal microbiome may be the result of the different environments and diets to which the examined animals were exposed [45, 46]. Also, the use of nanopore long-read and not old-school V3-V4 16S rRNA gene sequencing might have contributed to differences from what has been observed before. Though, using full-length 16S rRNA gene sequences was shown to provide an analytical advantage [47]. Figure 3A showed that the abundance of Firmicutes decreased in favour of Bacteroidetes 21 days after the end of the conventional cefquinome treatment. Bacteroidetes members are known to negatively correlate with body weight in several mammals, including pigs [48, 49]. This means that administration of cefquinome might result in leaner pigs and thus a higher feed conversion ratio. In the current study, the weight of the pigs was not assessed, so this could not be confirmed.

Both ceftiofur and cefquinome treatment resulted in an overall increase in members of the Proteobacteria phylum in comparison to the control (Figure 4). Especially between the 7 dpt and the 21 dpt sampling point, a significant log_2_ fold change associated with an increase in Proteobacteria members was detected. This observed increase in relative abundance of Proteobacteria only after treatment with antimicrobials might be caused by the ARG carrying nature of this phylum. It has been reported before that antimicrobial treatment causes an increase in Proteobacteria and enriches the ARGs in this phylum, driving the ARG burden only further [50, 51]. Furthermore, the Proteobacteria phylum contains several potential opportunistic pathogens, such as *Escherichia* and *Helicobacter*. The increase in this phylum could, therefore, indicate a negative impact on overall gut health caused by antimicrobial treatment [52].

Only treatment with cefquinome increased some samples in *Elusimicrobium* 21 dpt. Members of the Elusimicrobia phylum are capable of producing H_2_, and are involved in the fermentative function of the gut. However, it is not clear whether or not their presence is beneficial for the host’s health [53, 54]. Since only a small subset of samples in the cefquinome group exhibited this increase, further research is required to investigate whether this finding is caused by cefquinome treatment or an observation of coincidence.

Interestingly, an increase in two members of the PVC (Planctomycetota, Verrucomicrobiota, and Chlamydiota) superphylum, Kiritimatiellaeota and Lentisphaerae, was observed only in the control group 21 dpt. These two phyla have been detected in the intestines of mammals, however, their function remains rather unclear [55]. For Lentisphaerae, their presence has been associated with a healthier gut microbiome in humans and weight gain in cattle [56]. An increase in these phyla might, therefore, suggest a healthier gut in comparison to the gut health of the pigs treated with either ceftiofur or cefquinome.

On family level, members of Lachnospiraceae (*Blautia*), Ruminococcaceae, Clostridiaceae (*Clostridium*) and Lactobacillaceae (*Lactobacillus*) were of relatively high abundance. The presence of these families is generally deemed beneficial during growth. The bacterial populations affiliated with Lachnospiraceae*,* Ruminococcaceae and Clostridiaceae can express enzymes that are associated with butyrate production and are thus important in the energy production necessary in growing pigs [57, 58]. Members of Lactobacillaceae, on the other hand, inhibit and prevent the growth of pathogens in the gastro-intestinal tract and some of its members can enhance the gut inflammatory response [59, 60].

It was demonstrated that by the end of treatment with cefquinome a decrease in *Lactobacillus* was observed associated with an increase in *Christensenella* and *Oscillibacter*. *Lactobacillus* is considered an important member of the gut microbiome and is often applied as a probiotic in pig industry [61]. Nevertheless, the presence of *Christensenella* and *Oscillibacter* has been associated with leaner and healthier pigs that are more feed-efficient [62, 63]. Thus overall, this change does not imply a detrimental impact on the piglet’s health.

A remarkable finding was the decrease of *Clostridium* members in the cefquinome group 21 days post-treatment, while in both control and ceftiofur group a steady increase over time was observed. The most abundant members were *C. tertium*, *C. disporicum* and *C. saudiense* (as identified by full-length 16S rRNA sequences), the first two being associated with serious cases of infection [64, 65]. Administration of cefquinome could, therefore, have a collateral beneficial impact on the gut *Clostridium* load. Further research should confirm or disprove this finding.

In our previous study, shotgun sequencing was applied to investigate both the effect of conventional ceftiofur administration on the microbiome as well as on the resistome in pig feces instead of 16S rRNA analysis [16]. Consequently, when comparing the microbiome results of this previous study and the current study, some discrepancies emerged. For example, with shotgun analysis, an increase in *Prevotella* was observed, while in this study no significant changes in *Prevotella* abundance could be detected. Furthermore, by including a control group in the current study, observations made in the previous study, such as the increase in *Blautia* abundance, could now be attributed to normal day-to-day fluctuations. Both shotgun sequencing and 16S rRNA gene sequencing have their own biases and pitfalls, which of these two methods is preferable over the other depends on the proposed research question [47, 66–68]. Typically, for taxonomic compositional analysis, 16S rRNA is considered the gold standard [69]. Therefore, the compositional results obtained in the current study are considered more useful to derive conclusions from than those from our previous study. However, it remains imperative to stress that when drawing conclusions from sequencing data, the results obtained via 16S rRNA gene sequencing (PCR amplification using conserved primers) and those from shotgun sequencing (no amplification) are not directly comparable and that it is important to be aware of the technique applied in each study. Still, differences in studies could also be attributed to the presence of ARGs on mobile genetic elements, such as plasmids. Our study showed the high prevalence of an *ErmB*-harbouring plasmid with repUS69 replicon in *Lactobacillus* and *Limosilactobacillus* species. Surprisingly, only two other plasmid borne ARGs could be identified, being *tetK* and *mcr4.2* within *Staphylococcus* and *Escherichia* classified reads, respectively. While the used library preparation (RBK-004) was shown to be favourable in studying plasmid sequences, sequencing throughput (241 326 (± 121.660) reads), sample complexity (i.e., fecal samples), read length N_50_ values (4023 (± 893) nucleotides), and clinical-focused replicon databases (e.g., PlasmidFinder) might pose a limitation to current investigations on plasmid sequences from shotgun sequencing data.

Overall, cefquinome treatment had a more substantial impact on the microbiome in comparison to ceftiofur treatment, with more than double the number of affected genera (18 vs 8, respectively). This was confirmed by the PCoA results (Figure 5B) that showed a clear impact of cefquinome treatment immediately post-treatment when compared to the microbiome composition of the control group.

The antibiotic resistome profiling revealed the presence of a diverse set of resistance genes in the fecal microbiome of pigs, even without the presence of antimicrobial pressure. Following other results in human, chicken, and pig samples, a relatively high abundance of ARGs associated with tetracycline and macrolide-lincosamide-streptogramin (MLS) was detected, with those directed against tetracyclines being the most abundant [70]. To the authors’ knowledge, there is no publicly available shotgun metagenomic data on the effect of either ceftiofur or cefquinome administration on the gastrointestinal resistome in pigs over time.

This study revealed a detectable increase in several ARGs immediately after treatment with either ceftiofur or cefquinome. After ceftiofur treatment, only one resistance gene was increased (*tetQ*). Conventional treatment with cefquinome, on the other hand, resulted in an increase of six ARGs, of which one was associated with resistance against beta-lactam antibiotics (i.e. *CfxA6*). The selection of non-associated ARGs, i.e. ARGs not directed against the applied antimicrobial, has been observed previously following treatment with antimicrobial agents belonging to different antimicrobial classes [71, 72]. A possible explanation for this observed collateral selection might be the association of the enriched genes with mobile genetic elements [73, 74]. This co-selection emphasizes the need to monitor the effect of antimicrobial treatment on multiple ARGs, even those not associated with the applied antimicrobial agent. As visually confirmed by the PCoA (Figure 7B), only cefquinome treatment exhibited a significant effect on the overall gut resistome in pigs immediately after treatment, mostly driven by the increase of the *mel* resistance gene.

Long-read shotgun sequencing allowed correlation of the ARGs with a significant log_2_ fold change to their corresponding genus. The most important resistance genes (i.e*. tetQ* and *CfxA6*) were mostly associated with either *Prevotella* and/or *Bacteroides*. This is in line with our previous study, where *Prevotella* and *Bacteroides* were the main genera of interest [16].

Following ceftiofur treatment, a selection of *tetQ* containing *Bacteroides* and of *CfxA6* containing *Prevotella* was observed. This increase of *CfxA6* on *Prevotella* is in contrast with our previous study, where an increase of *CfxA6* on *Bacteroides* instead of on *Prevotella* was observed. However, in the previous study, the level of *CfxA6* on *Prevotella* was already low to begin with, while in the current study it was the other way around, with *Bacteroides* containing only low levels of *CfxA6* at the start of the experiment. Moreover, our preliminary study was composed of fewer data points. Combining the results of the previous study and the current study, it can be concluded that rather the presence of the ARGs on a certain genus influences its selection than that the observed selection is the result of the presence of the specific genus.

Another remarkable finding caused by ceftiofur treatment was the appearance of *bla*_*TEM-1*_ associated *E. coli* (as determined by full-length 16S rRNA gene)*.* Throughout the study an increase in *Escherichia* and an appearance of *bla*_*TEM-1*_ in the samples taken 7 and 21 dpt was observed (not significant). It is known that the majority of *E. coli*’s carry a *bla*_*TEM-1*_ resistance gene and that later generations (i.e. the 4^th^ and 5^th^ generation) of cephalosporins, such as cefquinome, are not affected by the *bla*_*TEM-1*_ beta-lactamases [75]. Therefore, it could be that the inability to detect this gene in the before and end of treatment ceftiofur samples is rather caused by the low abundance of *E. coli* (± 0.3%) at the beginning of the experiment than by the true absence of *bla*_*TEM-1*_, and that cefquinome treatment does not impact its prevalence, hence no detection of *bla*_*TEM-1*_ associated *E. coli* in the cefquinome samples. The increase in both *bla*_*TEM-1*_ and *Escherichia* following ceftiofur treatment suggests a selection because of this administration. It would be interesting to investigate this finding further, since, although *bla*_*TEM-1*_ is not considered an ESBL, it can be transformed into an ESBL through mutations of the amino acid sequence around its active site [76]. The increase of *bla*_*TEM-1*_ and *CfxA* following the intramuscular administration of ceftiofur has been observed before in cattle treated with a combination of intramuscularly administered ceftiofur and intramammary administered cefquinome. In the same study on cattle, also a decrease in *Clostridium* members was observed, which might be caused by cefquinome, as observed in our study in pigs [77].

In contrast to the results of the differential abundance analysis and the PCoA (Figure 7B) from the cefquinome treatment group, correlating the ARGs of interest with their corresponding genera did not result in notable findings. Some increases and decreases were observed but did not seem to be of any importance. For example, *CfxA6*, the only cephalosporin-specific resistance gene that showed a significant log_2_ fold change, did not exhibit the same alterations on *Prevotella* as it did following ceftiofur treatment. It can, therefore, be concluded that cefquinome had a more general impact on the resistome instead of a more selective impact, as was observed for ceftiofur.

In general, the data suggests that the impact on gut resistome level for both cefquinome and ceftiofur is the most pronounced immediately following treatment (ET) with a return to the same levels as the control group by 21 days post-treatment. Further research is required to evaluate the functionality of the resistome (i.e. the gene expression), since it has been reported that only around 60% of all identified ARGs get expressed, and thus examining the composition of the resistome only shows a part of the impact of antimicrobial administration [78]. Also, the impact on other AMR mechanisms (i.e*.* point mutations within rRNA and/or *ParC/GyrA* genes) should be addressed as the landscape of acquired resistance is highly complex [79].

In this study, we investigated the impact of conventional treatment with either ceftiofur or cefquinome on the porcine gut microbiome and resistome in clinically healthy pigs, by applying a combination of 16S rRNA gene and shotgun sequencing. Our results suggest that the administration of cefquinome has a more substantial impact on the microbiome and general resistome of porcine fecal samples in comparison to the administration of ceftiofur. However, ceftiofur treatment seems to have a more specific impact on resistome alteration (i.e. selection of cephalosporin-specific associated ARGs). These findings provide more information on the collateral effect of the conventional treatment schedule of these important antimicrobials and can be used to formulate well-founded treatment decisions in the future.

## Supplementary Information

**Additional file 1: Overview of the 16S rRNA sequencing results for all samples.** 16S rRNA sequencing results following either ceftiofur treatment: 3 mg.kg^−1^ intramuscular, 3 consecutive days or cefquinome treatment: 2 mg.kg^−1^ intramuscular, 5 consecutive days. The raw sequencing datafiles are available on the European Nucleotide Archiveunder project PRJEB61541 and accessions ERS1494721-ERS14949789.

**Additional file 2: Overview of the shotgun sequencing results for all samples.** Shotgun sequencing results following either ceftiofur treatment: 3 mg.kg^−1^ intramuscular, 3 consecutive days or cefquinome treatment: 2 mg.kg^−1^ intramuscular, 5 consecutive days.

**Additional file 3: Rarefaction curves showing the taxon richness at genus level in function of the sequencing depth for each sample. A.** Rarefaction curves of the control group. **B. **Rarefaction curves of the ceftiofur group. **C.** Rarefaction curves of the cefquinome group. The grey dotted line depicts the cut-off used to correct for difference in sequencing depth during down-stream analysis. Following either ceftiofur treatment: 3 mg.kg^−1^ intramuscular, 3 consecutive days or cefquinome treatment: 2 mg.kg^−1^ intramuscular, 5 consecutive days.

**Additional file 4: Differential abundance analysis of the microbial genera in the porcine fecal samples. **Differential abundance analysis of the microbial genera that exhibited significantly increased/decreased abundances due to time and/or ceftiofur or cefquinome administration across different sampling points. Following either ceftiofur treatment: 3 mg.kg^−1^ intramuscular, 3 consecutive days or cefquinome treatment: 2 mg.kg^−1^ intramuscular, 5 consecutive days.

**Additional file 5: Pairwise comparison for Permutational Multivariate Analysis of Varianceusing Bray-Curtis dissimilarity index. **PERMANOVA of the ceftiofur groupto either the blank samples, to the control samples at the same sampling point or to the different timepoints within the ceftiofur group and of the cefquinome groupto either the control group, to the control samples at the same sampling point or to the different timepoints within the cefquinome group. Following either ceftiofur treatment: 3 mg.kg^−1^ intramuscular, 3 consecutive days or cefquinome treatment: 2 mg.kg^−1^ intramuscular, 5 consecutive days.

**Additional file 6: Correlation-coefficient analysis of the top 21 most abundant microbial genera in the porcine fecal samples.**

**Additional file 7: Pairwise comparison for Permutational Multivariate Analysis of Varianceusing Bray-Curtis dissimilarity index of the antimicrobial resistance genes. **PERMANOVA of the antimicrobial resistance genes of each treatment group at each sampling point in comparison to the control group. Following either ceftiofur treatment: 3 mg.kg^−1^ intramuscular, 3 consecutive days or cefquinome treatment: 2 mg.kg^−1^ intramuscular, 5 consecutive days.

**Additional file 8: Correlation-coefficient analysis of the antimicrobial resistance genes. **Correlation-coefficient analysis of the antimicrobial resistance genes in the porcine fecal samples with an R2 value greater than 0.4 at each sampling point.

**Additional file 9: Relative abundance of genera associated with the antimicrobial resistance genes. **Relative abundance of genera associated with the antimicrobial resistance genes that exhibit a significant log_2_-fold changefollowing either ceftiofur treatment: 3 mg.kg^−1^ intramuscular, 3 consecutive days or cefquinome treatment: 2 mg.kg^−1^ intramuscular, 5 consecutive days.

## Data Availability

The raw data supporting the conclusions of this article are available from the corresponding author on reasonable request.
