# Supplementary material for: Cefquinome shows a higher impact on the pig gut microbiome and resistome compared to ceftiofur
Source: Vet Res. 2023 Jun 6;54:45. doi: 10.1186/s13567-023-01176-8 (PMC10242799; doi:10.1186/s13567-023-01176-8)
Supplement: Supplementary file 1 — Additional file 1: Overview of the 16S rRNA sequencing results for all samples. 16S rRNA sequencing results following either ceftiofur treatment: 3 mg.kg−1 intramuscular, 3 consecutive days or cefquinome treatment: 2 mg.kg−1 intramuscular, 5 consecutive days. The raw sequencing datafiles are available on the European Nucleotide Archiveunder project PRJEB61541 and accessions ERS1494721-ERS14949789. [file 13567_2023_1176_MOESM1_ESM.docx]

**Additional file 1.** **Overview of the 16S rRNA sequencing results for all samples**. 16S rRNA sequencing results following either ceftiofur treatment: 3 mg.kg^−1^ intramuscular, 3 consecutive days or cefquinome treatment: 2 mg.kg^−1^ intramuscular, 5 consecutive days. (BT = Before Treatment, ET = End of Treatment, 7d = 7 days post-treatment, 21d = 21 days post-treatment, Cont = control, CT = ceftiofur, CQ = cefquinome).

| **ID** | **Sampling day** | **Group** | **Reads** | **N50** | **ID** | **Sampling day** | **Group** | **Reads** | **N50** |
| --- | --- | --- | --- | --- | --- | --- | --- | --- | --- |
| **4891** | BT | CQ | 107 851 | 1576 | **4925** | 7d | CQ | 104,721 | 1440 |
| **4892** | BT | CQ | 96 308 | 4581 | **4926** | 7d | CQ | 79 137 | 1435 |
| **4893** | BT | CQ | 97 492 | 4577 | **4927** | 7d | CQ | 88 957 | 1433 |
| **4894** | BT | CQ | 53 778 | 1577 | **4928** | 7d | CQ | 104 828 | 1434 |
| **4895** | BT | CQ | 77 147 | 1443 | **4929** | 7d | CQ | 106 658 | 1440 |
| **4896** | BT | CQ | 89 447 | 1577 | **4930** | 7d | CQ | 103 759 | 1443 |
| **4897** | BT | CT | 73 378 | 1589 | **4931** | 7d | CT | 113 697 | 1443 |
| **4898** | BT | CT | 79 221 | 1581 | **4932** | 7d | CT | 98 852 | 1441 |
| **4899** | BT | CT | 80 406 | 1577 | **4933** | 7d | CT | 114 584 | 1436 |
| **4900** | BT | CT | 100 379 | 1579 | **4934** | 7d | CT | 105 867 | 1436 |
| **4901** | BT | CT | 102 382 | 1574 | **4935** | 7d | CT | 32 398 | 1438 |
| **4902** | BT | CT | 117 777 | 1582 | **4936** | 7d | CT | 90 015 | 1440 |
| **4903** | BT | Cont | 83 733 | 1444 | **4937** | 7d | Cont | 64 693 | 1443 |
| **4904** | BT | Cont | 99 976 | 1576 | **4938** | 7d | Cont | 61 383 | 1433 |
| **4905** | BT | Cont | 97 159 | 1584 | **4939** | 7d | Cont | 59 023 | 1442 |
| **4906** | BT | Cont | 58 908 | 1594 | **4940** | 7d | Cont | 68 025 | 1436 |
| **4907** | BT | Cont | 64 183 | 1584 | **4941** | 7d | Cont | 66 108 | 1437 |
| **4908** | ET | CQ | 89 715 | 1577 | **4942** | 21d | CQ | 79 078 | 1437 |
| **4909** | ET | CQ | 83 250 | 1578 | **4943** | 21d | CQ | 71 548 | 1438 |
| **4910** | ET | CQ | 107 712 | 1583 | **4944** | 21d | CQ | 79 630 | 1435 |
| **4911** | ET | CQ | 107 264 | 1574 | **4945** | 21d | CQ | 75 200 | 1435 |
| **4912** | ET | CQ | 70 151 | 1577 | **4946** | 21d | CQ | 83 016 | 1438 |
| **4913** | ET | CQ | 89 215 | 1577 | **4947** | 21d | CQ | 70 374 | 1438 |
| **4914** | ET | CT | 98 327 | 1582 | **4948** | 21d | CT | 60 399 | 1439 |
| **4915** | ET | CT | 53 195 | 1445 | **4949** | 21d | CT | 66 382 | 1436 |
| **4916** | ET | CT | 65 880 | 1442 | **4950** | 21d | CT | 79 832 | 1432 |
| **4917** | ET | CT | 39 847 | 1438 | **4951** | 21d | CT | 82 679 | 1436 |
| **4918** | ET | CT | 71 684 | 1434 | **4952** | 21d | CT | 85 597 | 1435 |
| **4919** | ET | CT | 49 371 | 1450 | **4953** | 21d | CT | 82 545 | 1430 |
| **4920** | ET | Cont | 65 879 | 1446 | **4954** | 21d | Cont | 88 099 | 1435 |
| **4921** | ET | Cont | 97 483 | 1433 | **4955** | 21d | Cont | 86 217 | 1434 |
| **4922** | ET | Cont | 76 551 | 1449 | **4956** | 21d | Cont | 72 884 | 1433 |
| **4923** | ET | Cont | 63 983 | 1441 | **4957** | 21d | Cont | 71 273 | 1438 |
| **4924** | ET | Cont | 60 151 | 1441 | **4958** | 21d | Cont | 79 376 | 1440 |
