# Supplementary material for: Cefquinome shows a higher impact on the pig gut microbiome and resistome compared to ceftiofur
Source: Vet Res. 2023 Jun 6;54:45. doi: 10.1186/s13567-023-01176-8 (PMC10242799; doi:10.1186/s13567-023-01176-8)
Supplement: Supplementary file 2 — Additional file 2: Overview of the shotgun sequencing results for all samples. Shotgun sequencing results following either ceftiofur treatment: 3 mg.kg−1 intramuscular, 3 consecutive days or cefquinome treatment: 2 mg.kg−1 intramuscular, 5 consecutive days. [file 13567_2023_1176_MOESM2_ESM.docx]

**Additional file 2.** **Overview of the shotgun sequencing results for all samples.** Shotgun sequencing results following either ceftiofur treatment: 3 mg.kg^−1^ intramuscular, 3 consecutive days or cefquinome treatment: 2 mg.kg^−1^ intramuscular, 5 consecutive days. (BT = Before Treatment, ET = End of Treatment, 7d = 7 days post-treatment, 21d = 21 days post-treatment, Cont = control, CT = ceftiofur, CQ = cefquinome).

| **ID** | **Sampling day** | **Group** | **Reads** | **N50** | **ID** | **Sampling day** | **Group** | **Reads** | **N50** |
| --- | --- | --- | --- | --- | --- | --- | --- | --- | --- |
| **4891** | BT | CQ | 347 434 | 4152 | **4925** | 7d | CQ | 48 191 | 2485 |
| **4892** | BT | CQ | 306 737 | 4900 | **4926** | 7d | CQ | 199 763 | 4325 |
| **4893** | BT | CQ | 421 213 | 5282 | **4927** | 7d | CQ | 201 575 | 2376 |
| **4894** | BT | CQ | 423 024 | 5044 | **4928** | 7d | CQ | 242 928 | 5302 |
| **4895** | BT | CQ | 359 026 | 3808 | **4929** | 7d | CQ | 243 907 | 2905 |
| **4896** | BT | CQ | 308 314 | 3779 | **4930** | 7d | CQ | 297 371 | 5432 |
| **4897** | BT | CT | 399 762 | 3897 | **4931** | 7d | CT | 274 840 | 3083 |
| **4898** | BT | CT | 242 513 | 2358 | **4932** | 7d | CT | 203 095 | 4930 |
| **4899** | BT | CT | 275 534 | 4664 | **4933** | 7d | CT | 202 410 | 4826 |
| **4900** | BT | CT | 339 525 | 3841 | **4934** | 7d | CT | 197 756 | 4546 |
| **4901** | BT | CT | 496 295 | 3818 | **4935** | 7d | CT | 269 130 | 3469 |
| **4902** | BT | CT | 254 332 | 3365 | **4936** | 7d | CT | 271 580 | 4444 |
| **4903** | BT | Cont | 347 486 | 4152 | **4937** | 7d | Cont | 292 903 | 5497 |
| **4904** | BT | Cont | 306 742 | 4901 | **4938** | 7d | Cont | 290 867 | 4647 |
| **4905** | BT | Cont | 421 199 | 5281 | **4939** | 7d | Cont | 300 977 | 4935 |
| **4906** | BT | Cont | 422 969 | 5043 | **4940** | 7d | Cont | 273 450 | 4650 |
| **4907** | BT | Cont | 359 065 | 3807 | **4941** | 7d | Cont | 342 493 | 4058 |
| **4908** | ET | CQ | 138 033 | 4701 | **4942** | 21d | CQ | 389 331 | 3583 |
| **4909** | ET | CQ | 399 776 | 3895 | **4943** | 21d | CQ | 180 074 | 3937 |
| **4910** | ET | CQ | 242 477 | 2356 | **4944** | 21d | CQ | 234 937 | 3325 |
| **4911** | ET | CQ | 275 535 | 4662 | **4945** | 21d | CQ | 238 074 | 3164 |
| **4912** | ET | CQ | 339 527 | 3842 | **4946** | 21d | CQ | 373 014 | 2783 |
| **4913** | ET | CQ | 496 300 | 3817 | **4947** | 21d | CQ | 274 994 | 3052 |
| **4914** | ET | CT | 254 274 | 3364 | **4948** | 21d | CT | 207 307 | 4411 |
| **4915** | ET | CT | 223 894 | 3137 | **4949** | 21d | CT | 166 933 | 3589 |
| **4916** | ET | CT | 380 319 | 5245 | **4950** | 21d | CT | 65 142 | 4964 |
| **4917** | ET | CT | 123 277 | 4481 | **4951** | 21d | CT | 73 505 | 3790 |
| **4918** | ET | CT | 61 541 | 4348 | **4952** | 21d | CT | 64 548 | 3778 |
| **4919** | ET | CT | 109 494 | 4146 | **4953** | 21d | CT | 139 003 | 4374 |
| **4920** | ET | Cont | 82 026 | 3200 | **4954** | 21d | Cont | 65 066 | 5655 |
| **4921** | ET | Cont | 48 729 | 1922 | **4955** | 21d | Cont | 41 646 | 2386 |
| **4922** | ET | Cont | 48 914 | 3467 | **4956** | 21d | Cont | 138 501 | 4892 |
| **4923** | ET | Cont | 61 493 | 3338 | **4957** | 21d | Cont | 122 006 | 3901 |
| **4924** | ET | Cont | 101 454 | 4992 | **4958** | 21d | Cont | 64 634 | 3100 |
