# Supplementary material for: Cefquinome shows a higher impact on the pig gut microbiome and resistome compared to ceftiofur
Source: Vet Res. 2023 Jun 6;54:45. doi: 10.1186/s13567-023-01176-8 (PMC10242799; doi:10.1186/s13567-023-01176-8)
Supplement: Supplementary file 3 — Additional file 3: Rarefaction curves showing the taxon richness at genus level in function of the sequencing depth for each sample. A. Rarefaction curves of the control group. B. Rarefaction curves of the ceftiofur group. C. Rarefaction curves of the cefquinome group. The grey dotted line depicts the cut-off used to correct for difference in sequencing depth during down-stream analysis. Following either ceftiofur treatment: 3 mg.kg−1 intramuscular, 3 consecutive days or cefquinome treatment: 2 mg.kg−1 intramuscular, 5 consecutive days. [file 13567_2023_1176_MOESM3_ESM.docx]

**
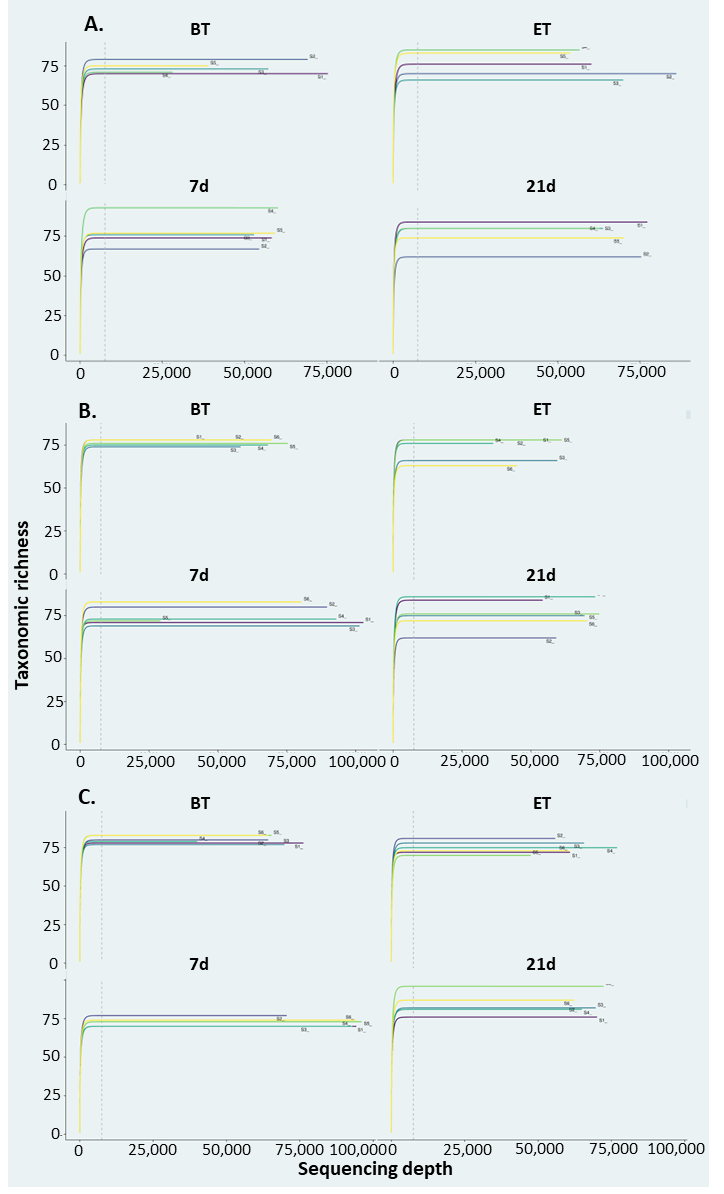
**

**Additional file 3.** **Rarefaction curves showing the taxon richness at genus level in function of the sequencing depth for each sample.** **A.** Rarefaction curves of the control group. **B.** Rarefaction curves of the ceftiofur group. **C.** Rarefaction curves of the cefquinome group. The grey dotted line depicts the cut-off used to correct for difference in sequencing depth during down-stream analysis. Following either ceftiofur treatment: 3 mg.kg^−1^ intramuscular, 3 consecutive days or cefquinome treatment: 2 mg.kg^−1^ intramuscular, 5 consecutive days. (BT = Before Treatment, ET = End of Treatment, 7d = 7 days post-treatment, 21d = 21 days post-treatment, Cont = control, CT = ceftiofur, CQ = cefquinome).
