# Supplementary material for: Cefquinome shows a higher impact on the pig gut microbiome and resistome compared to ceftiofur
Source: Vet Res. 2023 Jun 6;54:45. doi: 10.1186/s13567-023-01176-8 (PMC10242799; doi:10.1186/s13567-023-01176-8)
Supplement: Supplementary file 4 — Additional file 4: Differential abundance analysis of the microbial genera in the porcine fecal samples. Differential abundance analysis of the microbial genera that exhibited significantly increased/decreased abundances due to time and/or ceftiofur or cefquinome administration across different sampling points. Following either ceftiofur treatment: 3 mg.kg−1 intramuscular, 3 consecutive days or cefquinome treatment: 2 mg.kg−1 intramuscular, 5 consecutive days. [file 13567_2023_1176_MOESM4_ESM.docx]

**Additional file 4.** **Differential abundance analysis of the microbial genera in the porcine fecal samples.** Differential abundance analysis of the microbial genera that exhibited significantly increased/decreased abundances due to time and/or ceftiofur or cefquinome administration across different sampling points (FDR-corrected *P*-value < 0.10). Following either ceftiofur treatment: 3 mg.kg^−1^ intramuscular, 3 consecutive days or cefquinome treatment: 2 mg.kg^−1^ intramuscular, 5 consecutive days. (ET = End of Treatment, 7d = 7 days post-treatment, 21d = 21 days post-treatment, Cont = control, CT = ceftiofur, CQ = cefquinome).

ET_Cont vs 7d_Cont

| **Genus** | **baseMean** | **Log_2_FoldChange** | **q-value** | **Phylum** |
| --- | --- | --- | --- | --- |
| **Prevotellamassilia** | 41.37 | 23.29 | 0.00 | Bacteroidetes |
| **Paraprevotella** | 25.64 | 22.88 | 0.00 | Bacteroidetes |
| **Solobacterium** | 27.11 | 22.95 | 0.00 | Firmicutes |
| **Anaerobacterium** | 35.74 | -8.58 | 0.09 | Firmicutes |

7d_Cont vs 21d_Cont

| **Genus** | **baseMean** | **Log_2_FoldChange** | **q-value** | **Phylum** |
| --- | --- | --- | --- | --- |
| **Subdoligranulum** | 55.09 | 9.36 | 0.06 | Firmicutes |
| **Pseudoflavonifractor** | 187.12 | -1.12 | 0.09 | Firmicutes |
| **Megasphaera** | 544.95 | 5.91 | 0.10 | Firmicutes |
| **Gemmiger** | 1154.12 | 1.67 | 0.09 | Firmicutes |
| **Anaerostipes** | 274.79 | 1.21 | 0.09 | Firmicutes |
| **Blautia** | 2587.04 | 0.99 | 0.09 | Firmicutes |
| **Alloprevotella** | 22.34 | 8.06 | 0.09 | Bacteroidetes |
| **Paludicola** | 19.16 | 7.84 | 0.09 | Firmicutes |
| **Anaerotignum** | 15.81 | 7.56 | 0.09 | Firmicutes |
| **Ethanoligenens** | 20.53 | -7.64 | 0.09 | Firmicutes |
| **Kiritimatiella** | 35.25 | -8.42 | 0.09 | Kiritimatiellaeota |
| **Unclassified Lentisphaerae** | 61.72 | -9.22 | 0.06 | Lentisphaerae |
| **Helicobacter** | 18.25 | 7.77 | 0.09 | Proteobacteria |
| **Streptococcus** | 112.83 | -22.91 | 0.00 | Firmicutes |
| **Casaltella** | 18.40 | -7.48 | 0.09 | Firmicutes |

ET_Cont vs 21d_Cont

| **Genus** | **baseMean** | **Log_2_FoldChange** | **q-value** | **Phylum** |
| --- | --- | --- | --- | --- |
| **Oribacterium** | 32.20 | 23.31 | 0.00 | Firmicutes |
| **Kiritimatiella** | 35.72 | -8.39 | 0.04 | Kiritimatiellaeota |
| **Caldicoprobacter** | 50.09 | -8.88 | 0.03 | Firmicutes |
| **Frisingicoccus** | 131.94 | -10.28 | 0.00 | Firmicutes |
| **Megasphaera** | 878.65 | 6.56 | 0.01 | Firmicutes |
| **Anaerostipes** | 296.42 | 1.32 | 0.07 | Firmicutes |
| **Anaerobutyricum** | 748.80 | 1.03 | 0.03 | Firmicutes |
| **Bariatricus** | 65.75 | -9.28 | 0.02 | Firmicutes |
| **Prevotella** | 1359.64 | 1.30 | 0.06 | Bacteroidetes |
| **Christensenella** | 1863.75 | -1.19 | 0.06 | Firmicutes |
| **Roseburia** | 2285.79 | 2.49 | 0.01 | Firmicutes |
| **Blautia** | 2948.46 | 1.21 | 0.04 | Firmicutes |
| **Gemmiger** | 1280.06 | 1.84 | 0.06 | Firmicutes |
| **Paludicola** | 17.87 | 7.71 | 0.06 | Firmicutes |
| **Muribaculum** | 16.84 | 7.62 | 0.06 | Bacteroidetes |
| **Prevotellamassilia** | 44.89 | 23.77 | 0.00 | Bacteroidetes |
| **Alloprevotella** | 34.30 | 8.65 | 0.00 | Bacteroidetes |
| **Casaltella** | 18.86 | -7.47 | 0.06 | Firmicutes |
| **Ethanoligenens** | 20.72 | -7.61 | 0.06 | Firmicutes |
| **Solobacterium** | 32.46 | 23.33 | 0.00 | Firmicutes |
| **Anaerobacterium** | 30.48 | -8.17 | 0.05 | Firmicutes |
| **Streptococcus** | 116.21 | -24.09 | 0.00 | Firmicutes |
| **Unclassified Lentisphaerae** | 62.62 | -9.20 | 0.02 | Lentisphaerae |

ET_CT vs 7d_CT

| **Genus** | **baseMean** | **Log_2_FoldChange** | **q-value** | **Phylum** |
| --- | --- | --- | --- | --- |
| **Anaerotruncus** | 26.06 | 8.47 | 0.01 | Firmicutes |
| **Gemmiger** | 1366.89 | 1.81 | 0.00 | Firmicutes |
| **Parabacteroides** | 19.61 | -21.78 | 0.00 | Bacteroidetes |
| **Anaerocella** | 89.48 | -23.35 | 0.00 | Bacteroidetes |
| **Acidaminococcus** | 38.40 | -22.71 | 0.00 | Firmicutes |
| **Enterococcus** | 28.14 | -22.29 | 0.00 | Firmicutes |
| **Anaerobacterium** | 38.66 | -8.43 | 0.01 | Firmicutes |
| **Anaerocolumna** | 14.00 | -21.31 | 0.00 | Firmicutes |

7d_CT vs 21d_CT

| **Genus** | **baseMean** | **Log_2_FoldChange** | **q-value** | **Phylum** |
| --- | --- | --- | --- | --- |
| **Romboutsia** | 934.80 | -1.79 | 0.06 | Firmicutes |
| **Intestinibacter** | 428.75 | -0.94 | 0.09 | Firmicutes |
| **Terrisporobacter** | 3640.13 | -1.55 | 0.06 | Firmicutes |
| **Barnesiella** | 16.53 | 22.43 | 0.00 | Bacteroidetes |
| **Kineothrix** | 52.81 | 8.99 | 0.02 | Firmicutes |
| **Turicibacter** | 18.36 | -22.19 | 0.00 | Firmicutes |
| **Sterolibcaterium** | 20.35 | -7.93 | 0.09 | Proteobacteria |
| **Thiobacillus** | 72.94 | -9.77 | 0.00 | Proteobacteria |
| **Gallionella** | 80.11 | -9.91 | 0.00 | Proteobacteria |

ET_CT vs 21d_CT

| **Genus** | **baseMean** | **Log_2_FoldChange** | **q-value** | **Phylum** |
| --- | --- | --- | --- | --- |
| **Romboutsia** | 596.10 | -3.88 | 0.00 | Firmicutes |
| **Barnesiella** | 24.78 | 8.26 | 0.01 | Bacteroidetes |
| **Faecalicatena** | 175.38 | -0.92 | 0.01 | Firmicutes |
| **Catenibacterium** | 15.12 | 22.65 | 0.00 | Firmicutes |
| **Kineothrix** | 86.05 | 10.06 | 0.00 | Firmicutes |
| **Neglecta** | 150.10 | 1.90 | 0.08 | Firmicutes |
| **Coprococcus** | 1160.55 | -0.98 | 0.06 | Firmicutes |
| **Anaerobutyricum** | 539.57 | 0.66 | 0.06 | Firmicutes |
| **Gemmiger** | 1210.55 | 1.84 | 0.01 | Firmicutes |
| **Paludicola** | 9.47 | 6.87 | 0.09 | Firmicutes |
| **Prevotellamassilia** | 13.86 | 22.53 | 0.00 | Bacteroidetes |
| **Dialister** | 15.62 | 22.69 | 0.00 | Firmicutes |
| **Alloprevotella** | 26.42 | 23.41 | 0.00 | Bacteroidetes |
| **Escherichia** | 198.85 | -5.32 | 0.00 | Proteobacteria |
| **Parabacteroides** | 53.24 | -9.05 | 0.00 | Bacteroidetes |
| **Anaerocella** | 141.50 | -24.68 | 0.00 | Bacteroidetes |
| **Gallionella** | 62.45 | -9.28 | 0.00 | Proteobacteria |
| **Bacteroides** | 20.62 | -21.87 | 0.00 | Bacteroidetes |
| **Enterococcus** | 27.41 | -22.40 | 0.00 | Firmicutes |
| **Sterolibacterium** | 16.23 | -7.34 | 0.07 | Proteobacteria |
| **Anaerobacterium** | 30.26 | -8.24 | 0.00 | Firmicutes |
| **Anaerocolumna** | 26.72 | -8.06 | 0.01 | Firmicutes |
| **Thiobacillus** | 57.18 | -9.15 | 0.00 | Proteobacteria |

ET_CQ vs 7d_CQ

| **Genus** | **baseMean** | **Log_2_FoldChange** | **q-value** | **Phylum** |
| --- | --- | --- | --- | --- |
| **Frisingicoccus** | 49.54 | -8.94 | 0.02 | Firmicutes |
| **Helicobacter** | 13.94 | 7.31 | 0.08 | Proteobacteria |
| **Robinsoniella** | 40.97 | 23.84 | 0.00 | Firmicutes |
| **Butyrivibrio** | 166.32 | -10.69 | 0.00 | Firmicutes |
| **Phascolarctobacterium** | 554.18 | -1.24 | 0.01 | Firmicutes |
| **Coprococcus** | 982.67 | -2.57 | 0.00 | Firmicutes |
| **Bariatricus** | 231.32 | 11.37 | 0.00 | Firmicutes |
| **Oscillibacter** | 2680.88 | 0.81 | 0.08 | Firmicutes |
| **Intestinibacillus** | 492.43 | 2.89 | 0.00 | Firmicutes |
| **Lactobacillus** | 6724.00 | -2.60 | 0.08 | Firmicutes |
| **Anaerotignum** | 15.28 | -21.56 | 0.00 | Firmicutes |
| **Cellulosilyticum** | 38.45 | 8.78 | 0.02 | Firmicutes |
| **Peptococcus** | 115.57 | -10.16 | 0.00 | Firmicutes |
| **Anaerotaenia** | 34.85 | -8.43 | 0.03 | Firmicutes |
| **Ruminiclostridium** | 18.63 | 20.68 | 0.00 | Firmicutes |
| **Pelobacter** | 15.06 | -21.54 | 0.00 | Proteobacteria |
| **Enterococcus** | 35.80 | -22.74 | 0.00 | Firmicutes |
| **Anaerobacterium** | 37.62 | -8.54 | 0.01 | Firmicutes |

7d_CQ vs 21d_CQ

| **Genus** | **baseMean** | **Log_2_FoldChange** | **q-value** | **Phylum** |
| --- | --- | --- | --- | --- |
| **Megasphaera** | 431.73 | 4.72 | 0.08 | Firmicutes |
| **Gemmiger** | 756.27 | 1.85 | 0.06 | Firmicutes |
| **Butyricicoccus** | 1485.43 | 1.51 | 0.06 | Firmicutes |
| **Robinsoniella** | 55.77 | -9.28 | 0.00 | Firmicutes |
| **Bacteroides** | 39.79 | -8.79 | 0.03 | Bacteroidetes |
| **Bariatricus** | 46.69 | -9.02 | 0.02 | Firmicutes |
| **Pelobacter** | 14.69 | 22.37 | 0.00 | Proteobacteria |
| **Helicobacter** | 49.29 | -9.10 | 0.02 | Proteobacteria |
| **Fibrobacter** | 88.35 | -23.50 | 0.00 | Fibrobacteres |
| **Turicibacter** | 17.35 | -21.73 | 0.00 | Firmicutes |
| **Alcaligenes** | 18.49 | -21.96 | 0.00 | Proteobacteria |
| **Sterolibacterium** | 42.57 | -23.12 | 0.00 | Proteobacteria |
| **Thiobacillus** | 50.29 | -23.35 | 0.00 | Proteobacteria |
| **Gallionella** | 88.16 | -24.14 | 0.00 | Proteobacteria |
| **Falcatimonas** | 34.14 | -8.57 | 0.01 | Firmicutes |
| **Elusimicrobium** | 14.93 | -21.66 | 0.00 | Elusimicrobia |
| **Alistipes** | 32.06 | -8.48 | 0.03 | Bacteroidetes |
| **Acutalibacter** | 43.54 | -8.92 | 0.02 | Firmicutes |

ET_CQ vs 21d_CQ

| **Genus** | **baseMean** | **Log_2_FoldChange** | **q-value** | **Phylum** |
| --- | --- | --- | --- | --- |
| **Romboutsia** | 376.07 | -1.33 | 0.03 | Firmicutes |
| **Frisingicoccus** | 67.25 | -9.51 | 0.00 | Firmicutes |
| **Faecalicatena** | 188.00 | -1.23 | 0.00 | Firmicutes |
| **Dorea** | 302.03 | 4.78 | 0.03 | Firmicutes |
| **Butyrivibrio** | 102.59 | -10.11 | 0.00 | Firmicutes |
| **Hungateiclostridium** | 413.12 | -0.94 | 0.05 | Firmicutes |
| **Pseudobutyrivibrio** | 98.15 | -10.05 | 0.00 | Firmicutes |
| **Cuneatibacter** | 174.32 | 2.44 | 0.07 | Firmicutes |
| **Phascolarctobacterium** | 591.90 | -1.48 | 0.00 | Firmicutes |
| **Anaeromassilibacillus** | 304.82 | 2.69 | 0.00 | Firmicutes |
| **Neglecta** | 195.98 | 1.00 | 0.07 | Firmicutes |
| **Coprococcus** | 894.72 | -2.58 | 0.00 | Firmicutes |
| **Papillibacter** | 1805.20 | -0.97 | 0.08 | Firmicutes |
| **Faecalibacterium** | 955.33 | 2.25 | 0.01 | Firmicutes |
| **Anarobutyricum** | 557.31 | 0.81 | 0.07 | Firmicutes |
| **Oscillibacter** | 2199.17 | 1.22 | 0.00 | Firmicutes |
| **Unclassified Bacteroidetes** | 3598.86 | -1.02 | 0.06 | Bacteroidetes |
| **Intestinibacillus** | 389.17 | 6.06 | 0.00 | Firmicutes |
| **Gemmiger** | 1237.58 | 2.80 | 0.00 | Firmicutes |
| **Anaerotignum** | 20.85 | -7.82 | 0.03 | Firmicutes |
| **Cellulosilyticum** | 35.02 | 8.71 | 0.02 | Firmicutes |
| **Peptococcus** | 112.47 | -10.25 | 0.00 | Firmicutes |
| **Anaerotaenia** | 70.82 | -9.58 | 0.00 | Firmicutes |
| **Butyricimonas** | 55.15 | 9.37 | 0.00 | Bacteroidetes |
| **Ruminiclostridium** | 18.08 | 22.80 | 0.00 | Firmicutes |
| **Anaerocella** | 970.97 | -7.55 | 0.03 | Bacteroidetes |
| **Gallionella** | 76.89 | -23.94 | 0.00 | Proteobacteria |
| **Bacteroides** | 32.94 | -8.48 | 0.02 | Bacteroidetes |
| **Acetivibrio** | 11.95 | -7.01 | 0.06 | Firmicutes |
| **Fibrobacter** | 75.39 | -23.08 | 0.00 | Fibrobacteres |
| **Sterolibacterium** | 36.76 | -22.91 | 0.00 | Proteobaceria |
| **Anaerobacterium** | 35.18 | -8.57 | 0.00 | Firmicutes |
| **Anaerocolumna** | 21.22 | -22.15 | 0.00 | Firmicutes |
| **Alcaligenes** | 15.89 | -21.74 | 0.00 | Proteobacteria |
| **Thiobacillus** | 43.28 | -21.83 | 0.00 | Proteobacteria |
| **Turicibacter** | 17.34 | -21.87 | 0.00 | Firmicutes |
| **Falcatimonas** | 30.97 | -8.39 | 0.00 | Firmicutes |
| **Elusimicrobium** | 12.26 | -7.05 | 0.06 | Elusimicrobia |
| **Alistipes** | 26.68 | -8.17 | 0.03 | Bacteroidetes |
