# Supplementary material for: Cefquinome shows a higher impact on the pig gut microbiome and resistome compared to ceftiofur
Source: Vet Res. 2023 Jun 6;54:45. doi: 10.1186/s13567-023-01176-8 (PMC10242799; doi:10.1186/s13567-023-01176-8)
Supplement: Supplementary file 5 — Additional file 5: Pairwise comparison for Permutational Multivariate Analysis of Varianceusing Bray-Curtis dissimilarity index. PERMANOVA of the ceftiofur groupto either the blank samples, to the control samples at the same sampling point or to the different timepoints within the ceftiofur group and of the cefquinome groupto either the control group, to the control samples at the same sampling point or to the different timepoints within the cefquinome group. Following either ceftiofur treatment: 3 mg.kg−1 intramuscular, 3 consecutive days or cefquinome treatment: 2 mg.kg−1 intramuscular, 5 consecutive days. [file 13567_2023_1176_MOESM5_ESM.docx]

**Additional file 5.** **Pairwise comparison for Permutational Multivariate Analysis of Variance (PERMANOVA) using Bray-Curtis dissimilarity index.** PERMANOVA of the ceftiofur group (CT) to either the blank samples (*n* = 17, fecal samples of all pigs taken before treatment), to the control samples at the same sampling point or to the different timepoints within the ceftiofur group and of the cefquinome group (CQ) to either the control group (Cont), to the control samples at the same sampling point or to the different timepoints within the cefquinome group. Following either ceftiofur treatment: 3 mg.kg^−1^ intramuscular, 3 consecutive days or cefquinome treatment: 2 mg.kg^−1^ intramuscular, 5 consecutive days. (ET = End of Treatment, 7d = 7 days post-treatment, 21d = 21 days post-treatment).

|  | **SumsOfSqs** | **F-Model** | **R2** | ***p*-value** | **q-value*** |
| --- | --- | --- | --- | --- | --- |
| **Blank vs ET_CT** | 0.1965 | 3.2385 | 0.1336 | 0.0030 | **0.0090** |
| **Blank vs 7d_CT** | 0.2664 | 4.3956 | 0.1731 | 0.0010 | **0.0045** |
| **Blank vs 21d_CT** | 0.3226 | 5.3462 | 0.2029 | 0.0010 | **0.0045** |
| **ET_CT vs 7d_CT** | 0.0745 | 1.3231 | 0.1169 | 0.1848 | 0.2376 |
| **ET_CT vs 21d_CT** | 0.1710 | 3.6052 | 0.2346 | 0.0110 | **0.0248** |
| **7d_CT vs 21d_CT** | 0.0896 | 1.6109 | 0.1387 | 0.1698 | 0.2376 |
| **ET_Cont vs ET_CT** | 0.0528 | 0.7868 | 0.0804 | 0.6004 | 0.6004 |
| **7d_Cont vs 7d_CT** | 0.0689 | 1.1113 | 0.1099 | 0.3007 | 0.3383 |
| **21d_Cont vs 21d_CT** | 0.0971 | 1.5636 | 0.1480 | 0.1469 | 0.2376 |

*FDR-corrected *p*-value < 0.10

|  | **SumsOfSqs** | **F-Model** | **R2** | ***p*-value** | **q-value*** |
| --- | --- | --- | --- | --- | --- |
| **Blank vs ET_CQ** | 0.1519 | 2.6224 | 0.1110 | 0.0050 | **0.0075** |
| **Blank vs 7d_CQ** | 0.2356 | 3.5319 | 0.1440 | 0.0020 | **0.0036** |
| **Blank vs 21d_CQ** | 0.3871 | 5.953 | 0.2209 | 0.0020 | **0.0036** |
| **ET_CQ vs 7d_CQ** | 0.1777 | 2.804 | 0.219 | 0.0020 | **0.0036** |
| **ET_CQ vs 21d_CQ** | 0.2344 | 3.9141 | 0.2813 | 0.0020 | **0.0036** |
| **7d_CQ vs 21d_CQ** | 0.1568 | 2.004 | 0.1670 | 0.0639 | **0.0822** |
| **ET_Cont vs ET_CQ** | 0.2936 | 4.8414 | 0.3498 | 0.0020 | **0.0036** |
| **7d_Cont vs 7d_CQ** | 0.0834 | 1.0938 | 0.1084 | 0.3786 | 0.3786 |
| **21d_Cont vs 21d_CQ** | 0.1436 | 1/9658 | 0.1793 | 0.0889 | 0.1000 |

*FDR-corrected *p*-value < 0.10
