# Supplementary material for: Cefquinome shows a higher impact on the pig gut microbiome and resistome compared to ceftiofur
Source: Vet Res. 2023 Jun 6;54:45. doi: 10.1186/s13567-023-01176-8 (PMC10242799; doi:10.1186/s13567-023-01176-8)
Supplement: Supplementary file 6 — Additional file 6: Correlation-coefficient analysis of the top 21 most abundant microbial genera in the porcine fecal samples. [file 13567_2023_1176_MOESM6_ESM.docx]

**Additional file 6.** **Correlation-coefficient analysis of the top 21 most abundant microbial genera in the porcine fecal samples.**

**Ceftiofur**

|  | **Dim1** | **Dim2** | **R2** | **q-value*** |
| --- | --- | --- | --- | --- |
| **Clostridium** | -0.9136 | 0.4067 | 0.8083 | 0.002 |
| **Blautia** | 0.3242 | -0.9460 | 0.6624 | 0.002 |
| **Lactobacillus** | 0.6285 | 0.7778 | 0.9180 | 0.002 |
| **Ruminococcus** | 0.3345 | -0.9424 | 0.3231 | 0.002 |
| **Gemmiger** | 0.5012 | -0.8653 | 0.3243 | 0.002 |
| **Oscillibacter** | -0.5465 | -0.8374 | 0.4533 | 0.002 |
| **Faecalibacterium** | 0.6197 | -0.7848 | 0.2890 | 0.002 |
| **Dysosmobacter** | -0.9901 | 0.1403 | 0.3199 | 0.002 |
| **Sporobacter** | -0.9077 | -0.4197 | 0.2930 | 0.002 |
| **Unclassified Bacteroidetes** | -0.9859 | -0.1675 | 0.3640 | 0.002 |
| **Papillibacter** | -0.5635 | 0.8261 | 0.2556 | 0.002 |
| **Eubacterium** | 0.9041 | -0.4273 | 0.2651 | 0.004 |
| **Roseburia** | 0.9014 | -0.4330 | 0.2100 | 0.006 |
| **Terrisporobacter** | -0.9727 | 0.2321 | 0.1866 | 0.008 |
| **Christensenella** | -0.9456 | -0.3253 | 0.1607 | 0.016 |
| **Unclassified Lachnospiraceae** | 0.6349 | -0.7726 | 0.1852 | 0.016 |
| **Prevotella** | 0.4975 | -0.8675 | 0.1352 | 0.035 |
| **Mediterraneibacter** | -0.1419 | -0.9899 | 0.0899 | 0.140 |
| **Lacrimispora** | 0.2483 | -0.9687 | 0.0462 | 0.370 |
| **Coprococcus** | 0.9312 | 0.3644 | 0.0271 | 0.580 |
| **Butyricicoccus** | 0.9767 | 0.2147 | 0.0118 | 0.768 |

*FDR-corrected *p*-value **Cefquinome**

|  | **Dim1** | **Dim2** | **R2** | **q-value*** |
| --- | --- | --- | --- | --- |
| **Papillibacter** | 0.9977 | 0.0674 | 0.7606 | 0.002 |
| **Lactobacillus** | 0.1897 | 0.9818 | 0.7496 | 0.002 |
| **Blautia** | -0.9939 | -0.1102 | 0.6926 | 0.002 |
| **Faecalibacterium** | -0.9932 | 0.1168 | 0.4950 | 0.002 |
| **Roseburia** | -0.8470 | 0.5315 | 0.4940 | 0.002 |
| **Gemmiger** | -0.9972 | -0.0745 | 0.4372 | 0.002 |
| **Terrisporobacter** | -0.4531 | -0.8915 | 0.4228 | 0.002 |
| **Unclassified Bacteroidetes** | 0.9469 | -0.3215 | 0.4167 | 0.002 |
| **Dysosmobacter** | 0.7873 | -0.6166 | 0.3977 | 0.002 |
| **Sporobacter** | 0.7127 | -0.7015 | 0.3806 | 0.002 |
| **Christensenella** | 0.7793 | -0.6266 | 0.3727 | 0.002 |
| **Clostridium** | 0.0446 | -0.9990 | 0.3665 | 0.002 |
| **Eubacterium** | -0.7237 | 0.6901 | 0.3392 | 0.002 |
| **Oscillibacter** | -0.0196 | -0.9998 | 0.2904 | 0.003 |
| **Butyricicoccus** | -0.9948 | 0.1018 | 0.1723 | 0.008 |
| **Coprococcus** | -0.0373 | 0.9993 | 0.1110 | 0.066 |
| **Unclassified Lachnospiraceae** | 0.8428 | 0.5382 | 0.1051 | 0.074 |
| **Prevotella** | -0.9795 | 0.2014 | 0.1027 | 0.077 |
| **Lacrimispora** | 0.9832 | 0.1826 | 0.0794 | 0.161 |
| **Mediterraneibacter** | -0.8373 | -0.5468 | 0.0141 | 0.757 |
| **Ruminococcus** | -0.3667 | 0.9303 | 0.0029 | 0.934 |

*FDR-corrected p-value
