# Supplementary material for: Cefquinome shows a higher impact on the pig gut microbiome and resistome compared to ceftiofur
Source: Vet Res. 2023 Jun 6;54:45. doi: 10.1186/s13567-023-01176-8 (PMC10242799; doi:10.1186/s13567-023-01176-8)
Supplement: Supplementary file 7 — Additional file 7: Pairwise comparison for Permutational Multivariate Analysis of Varianceusing Bray-Curtis dissimilarity index of the antimicrobial resistance genes. PERMANOVA of the antimicrobial resistance genes of each treatment group at each sampling point in comparison to the control group. Following either ceftiofur treatment: 3 mg.kg−1 intramuscular, 3 consecutive days or cefquinome treatment: 2 mg.kg−1 intramuscular, 5 consecutive days. [file 13567_2023_1176_MOESM7_ESM.docx]

**Additional file 7.** **Pairwise comparison for Permutational Multivariate Analysis of Variance (PERMANOVA) using Bray-Curtis dissimilarity index of the antimicrobial resistance genes**. PERMANOVA of the antimicrobial resistance genes of each treatment group at each sampling point in comparison to the control group. Following either ceftiofusr treatment: 3 mg.kg^−1^ intramuscular, 3 consecutive days or cefquinome treatment: 2 mg.kg^−1^ intramuscular, 5 consecutive days. (ET = End of Treatment, 7d = 7 days post- treatment, 21d = 21 days post-treatment, Cont = controle, CT = Ceftiofur, CQ = Cefquinome).

|  | **SumsOfSqs** | **F-Model** | **R2** | ***p*-value** | **q-value*** |
| --- | --- | --- | --- | --- | --- |
| **ET_Cont vs ET_CT** | 0.0521 | 3.0926 | 0.2257 | 0.0679 | 0.1898 |
| **ET_Cont vs ET_CQ** | 0.1333 | 7.5138 | 0.4550 | 0.0120 | **0.0720** |
| **7d_Cont vs 7d_CT** | 0.0527 | 1.5618 | 0.1479 | 0.1658 | 0.2182 |
| **7d_Cont vs 7d_CQ** | 0.0670 | 1.5182 | 0.1443 | 0.1818 | 0.2182 |
| **21d_Cont vs 21d_CT** | 0.0397 | 1.1872 | 0.1165 | 0.2967 | 0.2967 |
| **21d_Cont vs 21d_CQ** | 0.0358 | 1.9448 | 0.1777 | 0.0949 | 0.1898 |

*FDR-corrected *p*-value < 0.10
