# Supplementary material for: Cefquinome shows a higher impact on the pig gut microbiome and resistome compared to ceftiofur
Source: Vet Res. 2023 Jun 6;54:45. doi: 10.1186/s13567-023-01176-8 (PMC10242799; doi:10.1186/s13567-023-01176-8)
Supplement: Supplementary file 8 — Additional file 8: Correlation-coefficient analysis of the antimicrobial resistance genes. Correlation-coefficient analysis of the antimicrobial resistance genes in the porcine fecal samples with an R2 value greater than 0.4 at each sampling point. [file 13567_2023_1176_MOESM8_ESM.docx]

**Additional file 8.** **Correlation-coefficient analysis of the antimicrobial resistance genes. Correlation**-coefficient analysis of the antimicrobial resistance genes in the porcine fecal samples with an R2 value greater than 0.4 at each sampling point.

**Before treatment**

|  | **Dim1** | **Dim2** | **R2** | **q-value*** |
| --- | --- | --- | --- | --- |
| ***tetQ*** | -0.9612 | -0.2757 | 0.9059 | 0.003 |
| ***CfxA6*** | -0.9192 | -0.3939 | 0.8569 | 0.003 |
| ***lsaB*** | -0.8507 | -0.5256 | 0.8250 | 0.003 |
| ***tetW*** | -0.3438 | -0.9390 | 0.7974 | 0.003 |
| ***mel*** | -0.9856 | 0.1691 | 0.7637 | 0.003 |
| ***tetX*** | -0.7182 | -0.6958 | 0.7283 | 0.003 |
| ***ErmF*** | -0.8655 | 0.5010 | 0.7280 | 0.003 |
| ***tetO*** | -0.9633 | 0.2683 | 0.7134 | 0.003 |
| ***APH(3')-IIIa*** | -0.8981 | -0.4397 | 0.7076 | 0.006 |
| ***ANT(6)-Ia*** | -0.9360 | 0.3519 | 0.6525 | 0.003 |
| ***lnuC*** | -0.9910 | -0.1340 | 0.5677 | 0.010 |
| ***tet(40)*** | -0.9988 | -0.0488 | 0.5627 | 0.011 |
| ***tet(W/N/W)*** | -0.7771 | -0.6294 | 0.5545 | 0.011 |
| ***SAT-4*** | -0.7320 | -0.6813 | 0.5329 | 0.008 |
| ***mdtG*** | -0.1526 | 0.9883 | 0.4971 | 0.011 |
| ***Escherichia coli mdfA*** | 0.0377 | 0.9993 | 0.4651 | 0.015 |
| ***APH(3')-VIIa*** | -0.5344 | -0.8452 | 0.4644 | 0.019 |
| ***Campylobacter coli chloramphenicol acetyltransferase*** | -0.5344 | -0.8452 | 0.4644 | 0.019 |
| ***evgS*** | -0.5344 | -0.8452 | 0.4644 | 0.019 |
| ***arnA*** | 0.0715 | 0.9974 | 0.4617 | 0.011 |
| ***emrK*** | 0.0715 | 0.9974 | 0.4617 | 0.011 |
| ***evgA*** | 0.0715 | 0.9974 | 0.4617 | 0.011 |
| ***mdtE*** | 0.0715 | 0.9974 | 0.4617 | 0.011 |
| ***mphB*** | 0.0715 | 0.9974 | 0.4617 | 0.011 |
| ***ErmB*** | 0.4012 | -0.9160 | 0.4612 | 0.024 |
| ***CfxA2*** | -0.9066 | -0.4220 | 0.4456 | 0.031 |
| ***cpxA*** | -0.3458 | 0.9383 | 0.4134 | 0.031 |
| ***acrD*** | -0.4564 | 0.8898 | 0.4119 | 0.031 |
| ***baeS*** | -0.4564 | 0.8898 | 0.4119 | 0.031 |
| ***vanRA*** | -0.7349 | -0.6782 | 0.4033 | 0.028 |

*FDR-corrected *p*-value

**End of treatment**

|  | **Dim1** | **Dim2** | **R2** | **q-value*** |
| --- | --- | --- | --- | --- |
| ***ErmB*** | -0.1432 | 0.9897 | 0.9477 | 0.003 |
| ***mel*** | -0.9426 | -0.3338 | 0.9429 | 0.003 |
| ***tetQ*** | -0.9871 | -0.1601 | 0.9053 | 0.003 |
| ***lsaB*** | -0.9800 | -0.1989 | 0.8897 | 0.003 |
| ***ErmF*** | -0.9293 | -0.3693 | 0.8692 | 0.003 |
| ***CfxA6*** | -0.9599 | -0.2805 | 0.7984 | 0.003 |
| ***lnuC*** | -0.7694 | -0.6388 | 0.7726 | 0.003 |
| ***tetW*** | -0.7340 | 0.6791 | 0.7703 | 0.003 |
| ***APH(3')-IIIa*** | -0.9343 | 0.3566 | 0.6993 | 0.003 |
| ***tet(40)*** | -0.9986 | 0.0521 | 0.6573 | 0.003 |
| ***tetO*** | -0.9846 | 0.1748 | 0.5943 | 0.003 |
| ***ErmA*** | -0.4673 | 0.8841 | 0.5648 | 0.003 |
| ***vanRG*** | -0.9487 | -0.3162 | 0.5634 | 0.007 |
| ***Escherichia_coli_mdfA*** | -0.5517 | -0.8341 | 0.4542 | 0.007 |
| ***mdtH*** | -0.5579 | -0.8299 | 0.4530 | 0.009 |
| ***mdtO*** | -0.5579 | -0.8299 | 0.4530 | 0.009 |
| ***mdtP*** | -0.5579 | -0.8299 | 0.4530 | 0.009 |
| ***CfxA2*** | -0.6735 | 0.7391 | 0.4230 | 0.017 |
| ***tetX*** | -0.8716 | -0.4902 | 0.4191 | 0.033 |
| ***tetA(P)*** | -0.6590 | -0.7521 | 0.4011 | 0.023 |

*FDR-corrected *p*-value

**7 days post-treatment**

|  | **Dim1** | **Dim2** | **R2** | **q-value*** |
| --- | --- | --- | --- | --- |
| ***ErmB*** | 0.9301 | -0.3673 | 0.9020 | 0.002 |
| ***tetW*** | 0.9477 | 0.3192 | 0.7558 | 0.002 |
| ***lnuC*** | 0.3184 | 0.9479 | 0.6590 | 0.002 |
| ***ErmF*** | 0.6781 | 0.7350 | 0.6540 | 0.002 |
| ***lsaB*** | 0.4744 | 0.8803 | 0.6388 | 0.004 |
| ***tetQ*** | 0.4591 | 0.8884 | 0.5989 | 0.002 |
| ***tet(40)*** | 0.7118 | 0.7024 | 0.5933 | 0.005 |
| ***mel*** | 0.3521 | 0.9359 | 0.5712 | 0.006 |
| ***ErmG*** | 0.9986 | -0.0531 | 0.4948 | 0.009 |
| ***cpxA*** | 0.9937 | -0.1123 | 0.4544 | 0.016 |
| ***ErmA*** | 0.9420 | -0.3357 | 0.4487 | 0.013 |

*FDR-corrected *p*-value

**21 days post-treatment**

|  | **Dim1** | **Dim2** | **R2** | **q-value*** |
| --- | --- | --- | --- | --- |
| ***mel*** | 0.7967 | -0.6044 | 0.8605 | 0.003 |
| ***tetQ*** | 0.9493 | -0.3145 | 0.8358 | 0.003 |
| ***CfxA6*** | 0.9695 | -0.2452 | 0.8270 | 0.003 |
| ***tetW*** | 0.8350 | 0.5502 | 0.7522 | 0.003 |
| ***tetA(P)*** | 0.5993 | -0.8006 | 0.7422 | 0.003 |
| ***ErmF*** | 0.9029 | -0.4299 | 0.7415 | 0.004 |
| ***lsaB*** | 0.8172 | 0.5764 | 0.7084 | 0.003 |
| ***ErmB*** | 0.6795 | 0.7337 | 0.6572 | 0.003 |
| ***ErmA*** | 0.7234 | 0.6904 | 0.6280 | 0.007 |
| ***mdtK*** | 0.1463 | -0.9892 | 0.6051 | 0.004 |
| ***tet(40)*** | 0.7649 | -0.6442 | 0.5995 | 0.003 |
| ***mdtN*** | 0.5888 | -0.8083 | 0.5971 | 0.004 |
| ***lnuA*** | 0.8220 | 0.5695 | 0.5755 | 0.004 |
| ***lnuC*** | 0.8018 | -0.5976 | 0.5707 | 0.004 |
| ***tetB(P)*** | 0.9123 | 0.4095 | 0.5615 | 0.011 |
| ***mdtO*** | 0.6117 | -0.7911 | 0.5283 | 0.010 |
| ***Escherichia coli ampC1 beta-lactamase*** | 0.0278 | -0.9996 | 0.5161 | 0.008 |
| ***kdpE*** | -0.0245 | -0.9997 | 0.4959 | 0.011 |
| ***LnuP*** | 0.8947 | -0.4467 | 0.4758 | 0.017 |
| ***tet(B)*** | 0.8634 | 0.5046 | 0.4528 | 0.056 |
| ***mdtF*** | -0.0209 | -0.9998 | 0.4497 | 0.023 |
| ***tetO*** | 0.7673 | -0.6413 | 0.4174 | 0.030 |
| ***mdtA*** | 0.5904 | -0.8071 | 0.4099 | 0.033 |
| ***mdtP*** | 0.9264 | -0.3765 | 0.4025 | 0.027 |
| ***eptA*** | -0.0536 | -0.9986 | 0.4005 | 0.034 |

*FDR-corrected *p*-value
