# Supplementary material for: Cefquinome shows a higher impact on the pig gut microbiome and resistome compared to ceftiofur
Source: Vet Res. 2023 Jun 6;54:45. doi: 10.1186/s13567-023-01176-8 (PMC10242799; doi:10.1186/s13567-023-01176-8)
Supplement: Supplementary file 9 — Additional file 9: Relative abundance of genera associated with the antimicrobial resistance genes. Relative abundance of genera associated with the antimicrobial resistance genes that exhibit a significant log2-fold changefollowing either ceftiofur treatment: 3 mg.kg−1 intramuscular, 3 consecutive days or cefquinome treatment: 2 mg.kg−1 intramuscular, 5 consecutive days. [file 13567_2023_1176_MOESM9_ESM.docx]

**Additional file 9.** **Relative abundance of genera associated with the antimicrobial resistance genes**. Relative abundance of genera associated with the antimicrobial resistance genes that exhibit a significant log_2_-fold change (shotgun sequencing data). Following either ceftiofur treatment: 3 mg.kg^−1^ intramuscular, 3 consecutive days or cefquinome treatment: 2 mg.kg^−1^ intramuscular, 5 consecutive days. (ET = End of Treatment, 7d = 7 days post-treatment, 21d = 21 days post-treatment, Cont = controle, CT = Ceftiofur, CQ = Cefquinome).

|  |  | **BT (*n* = 17)** | **ET** | **7d** | **21d** |
| --- | --- | --- | --- | --- | --- |
| **Prevotella** | **Control (*n* = 5)** | 7.55% | 4.56% | 6.06% | 3.28% |
|  | **Ceftiofur (*n* = 6)** |  | 7.91% | 5.38% | 6.27% |
|  | **Cefquinome (*n* = 6)** |  | 6.20% | 4.64% | 2.89% |
| **Bacteroides** | **Control (*n* = 5)** | 1.79% | 1.24% | 1.74% | 1.40% |
|  | **Ceftiofur (*n* = 6)** |  | 2.28% | 1.61% | 2.18% |
|  | **Cefquinome (*n* = 6)** |  | 1.42% | 1.66% | 1.42% |
| **Roseburia** | **Control (*n* = 5)** | 1.62% | 1.09% | 1.11% | 0.72% |
|  | **Ceftiofur (*n* = 6)** |  | 1.16% | 1.36% | 1.04% |
|  | **Cefquinome (*n* = 6)** |  | 1.06% | 1.20% | 1.11% |
| **Faecalibacterium** | **Control (*n* = 5)** | 2.94% | 2.17% | 2.41% | 1.84% |
|  | **Ceftiofur (*n* = 6)** |  | 3.71% | 1.55% | 2.25% |
|  | **Cefquinome (*n* = 6)** |  | 2.86% | 2.01% | 0.94% |
| **Clostridium** | **Control (*n* = 5)** | 4.15% | 5.19% | 5.70% | 4.32% |
|  | **Ceftiofur (*n* = 6)** |  | 3.88% | 4.62% | 4.83% |
|  | **Cefquinome (*n* = 6)** |  | 3.42% | 5.84% | 5.24% |
